# Ethosome-Based Colloidal Systems for Transdermal Delivery: The Role of Biosurfactant in Enhancing Stability and Efficacy

**DOI:** 10.3390/ma18235355

**Published:** 2025-11-27

**Authors:** Jagoda Chudzińska-Skorupinska, Agata Wawrzyńczak, Agnieszka Feliczak-Guzik

**Affiliations:** Department of Applied Chemistry, Faculty of Chemistry, Adam Mickiewicz University in Poznań, Uniwersytetu Poznańskiego 8, 61-614 Poznań, Poland; jagoda.chudzinska@amu.edu.pl (J.C.-S.); agata.wawrzynczak@amu.edu.pl (A.W.)

**Keywords:** active substance carriers, transdermal drug delivery systems, transethosomes, binary ethosomes, cold method, biosurfactant

## Abstract

The effectiveness of transdermal drug delivery is restricted by the barrier properties of the stratum corneum. Ethosomes, as vesicular carriers, offer a promising approach to enhance dermal bioavailability. This study aimed to optimize ethosome composition and preparation parameters to improve physicochemical stability and performance. The influence of alcohols (ethyl, n-butyl, n-propyl, isopropyl, tert-butyl), glycols (propylene glycol, ethylene glycol, 1,3-butanediol), and surfactants (Tween 80, Mirasoft^®^ SL L60) was systematically investigated. Stability was evaluated through zeta potential (ZP), polydispersity index (PDI), and hydrodynamic diameter (D*_h_*). The effects of phospholipid concentration and homogenization were also assessed. SEM imaging confirmed the spherical morphology of vesicles. The optimal formulation comprised 30% (*w*/*w*) ethanol, 2.5% (*w*/*w*) phospholipid, 10% (*w*/*w*) ethylene glycol, and 1.25% (*w*/*w*) Tween 80. A comparable mixed-surfactant system (0.625% *w*/*w*; 60% Tween 80 and 40% Mirasoft^®^ SL L60) exhibited similar stability, indicating that glycolipid-based biosurfactants can reduce conventional surfactant requirements. Homogenization significantly enhanced colloidal stability, lowering PDI from 0.366 to 0.083 and D*_h_* from 254 nm to 156 nm, evidencing decreased aggregation and improved size uniformity. Overall, formulation composition and processing conditions critically determine ethosome stability and transdermal delivery efficiency.

## 1. Introduction

Human skin is composed of several layers. The outermost layer is the epidermis (Stratum corneum), which, among others, serves as a protective barrier against the penetration of pathogenic microorganisms. The Stratum corneum consists of tightly packed dead cells—corneocytes [1]. Overcoming the epidermal barrier is necessary to deliver active substances into the deeper layers of the epidermis. Effective transport through the epidermis can proceed via various pathways. Three primary ways of active substance penetration through the outermost epidermal layer can be distinguished [2], namely, intercellular, transcellular, and follicular routes.

The intercellular route proceeds between corneocytes through the lipid layer and is preferred by lipophilic compounds. The transcellular route allows hydrophilic substances to penetrate directly through corneocytes. An alternative pathway, that bypasses corneocytes, is transport via hair follicles [2,3,4].

To enhance the efficiency of transdermal drug delivery system (TDDS), various types of carriers are used that support epidermal penetration. One well-known group of carriers comprises liposomes–spherical vesicles composed of a lipid bilayer and water. They can carry both lipophilic substances (within the lipid bilayer) and hydrophilic substances (within the vesicle’s aqueous core). However, liposomes have certain limitations, including low stability, fragility, leakiness, and possibility of the uncontrolled release of active substances [5,6]. Therefore, new solutions have been pursued to overcome these limitations. An alternative to conventional liposomes may be ethosomes, which are composed of a lipid bilayer and water but also contain alcohol, usually ethanol, in their structure [7]. These carriers may also include additional compounds that improve their stability and support skin penetration. Based on their composition, ethosomes can be classified into several types, namely classical, binary, and transethosomes [8,9]. Table 1 presents a comparison of the hydrodynamic diameter (D*_h_*) properties, zeta potential (ZP), and polydispersity index (PDI) of lipid-based carriers, namely liposomes, ethosomes, and exosomes.

Classical ethosomes are composed of lipids, water, and ethanol, whereas binary ethosomes include an additional alcohol, most often propylene glycol, alongside ethanol [16,17]. Typically, the ethanol concentration ranges from 20% (*w*/*w*) to 45% (*w*/*w*). It has been observed that, within this range, the hydrodynamic diameter of ethosomes increases as the ethanol concentration decreases [18]. Transethosomes are composed of lipids, water, ethanol, and a penetration enhancer (edge activator), most commonly a surfactant. Cholesterol is frequently added to enhance the stability of such carriers, as it prevents aggregation and positively influences the stability of the obtained vesicles. However, increasing the concentration of added cholesterol also increases the hydrodynamic diameter (D*_h_*) [19,20].

Natural phospholipids (such as phosphatidylcholine) possess negatively charged phosphate groups (–PO_4_^−^) in their polar heads, which are located on the exterior of the forming vesicle. In an aqueous environment, some of these groups remain ionized, thus creating a net negative charge on the vesicle surface [21]. The ethanol contained in ethosomes does not neutralize this charge; rather, it enhances the ionization of phosphate groups by lowering the dielectric constant of the medium (altering ion solubility). It also increases the availability of phosphate groups for interactions with water, thereby fluidizing the membrane, reducing lipid packing, and partially dehydrating the surface, which makes the effective charge (measured electrophoretically) more negative [9]. A strongly negative zeta potential (≥30 mV) prevents particle aggregation, as electrostatic repulsive forces exceed van der Waals forces. Consequently, ethosomes are often more stable than conventional liposomes [21,22].

Ethosomes can be prepared using several methods. The most important among them is the so-called cold method, which involves heating two phases to 30 °C: one phase being lipids dissolved in ethanol, and the other being the aqueous phase. The aqueous phase is then added to the lipid phase dropwise or slowly in a thin stream while stirring continuously. Stirring, most often using a magnetic stirrer, continues until vesicle formation is achieved. The hot method, in contrast, involves preparing a lipid dispersion in water as one phase and ethanol as the other. Both phases are heated to 40 °C, after which one phase is added dropwise to the other, with continuous stirring. The resulting ethosomes are stored under refrigeration [23,24,25].

According to the available literature, ethosomes have been prepared using ethanol, isopropanol, n-propanol, and glycerol [8]. Propylene glycol is commonly employed as a standard additive, although some studies report the use of polyethylene glycol (most often PEG 400) and 1,3-butanediol [23]. Regarding surfactants, the most frequently used surface-active compounds are Tween 80, as well as Span 60 and Span 80, which are nonionic surfactants [3]. However, to date, no comprehensive screening study has been conducted on the use of alcohols, glycols, and biosurfactants in ethosomal systems.

In line with the trend toward natural ingredients, raw materials derived from renewable sources are required. Consequently, in this study, the biosurfactant Mirasoft^®^ SL L60, introduced by the company Syensqo to the cosmetics market in 2022, was compared with the commonly used surfactant Tween 80 [26,27,28]. This glycolipid-based biosurfactant, developed from rapeseed oil and sugar, is produced via fermentation using natural yeast. It is a sophorolipid rich in lactones and exhibits effective solubilizing, cleansing, and emulsifying properties [29].

Therefore, the aim of this study is to optimize the preparation parameters of modern transdermal carriers, specifically ethosomes, including the evaluation of the impact of the concentrations of the phospholipid used (phosphatidylcholine), various aliphatic alcohols (ethanol, n-butanol, and n-propanol), branched alcohols (isopropanol, tert-butanol), glycols (ethylene glycol, propylene glycol, 1,3-butanediol), and surfactants (Tween 80, Mirasoft^®^ SL L60). Special emphasis has been placed on the stability evaluation for ethosomes prepared with surfactant mixtures containing a glycolipid-based biosurfactant obtained by yeast fermentation. The influence of the preparation method on the stability of the obtained ethosomes was also evaluated, based on measurements of zeta potential (ZP), hydrodynamic diameter (D*_h_*), and polydispersity index (PDI). During our study innovative formulations were developed that include both glycol and surfactant components, as well as mixtures containing biosurfactant, which to the best of our knowledge have not yet been described in the literature.

## 2. Materials and Methods

### 2.1. Materials

For the preparation of the formulations, the following substances were used: phospholipid–phosphatidylcholine (PHOSPHOLIPON 90G, Lipoid, Ludwigshafen, Germany), ethyl alcohol (P.P.H. ‘STANLAB’, Lublin, Poland, 96%), n-butanol (Eurochem BGD, Tarnów, Poland, analytical grade), isopropyl alcohol (P.P.H. ‘STANLAB’, analytical grade), n-propyl alcohol (Honeywell, Morris Plains, NJ, USA, analytical grade), tert-butanol (Honeywell, 99.5%), propylene glycol (Sigma-Aldrich, St. Louis, MO, USA, ≥99.5%), ethylene glycol (Hadron Scientific, Kielce, Poland, 99%), 1,3-butanediol (Fluka Analytical, Seelze, Germany, 99%), and surfactants, namely, Tween 80 (Sigma-Aldrich) and Mirasoft^®^ SL L60 (Syensqo, Brussels, Belgium).

In order to systematize the obtained formulations, the following labels were applied (Table 2).

Examples:

- P-x8T-M(1+4) denotes a sample prepared with the following composition: 10% (*w*/*w*) of propylene glycol, 0.625% (*w*/*w*) of Tween 80, 30% (*w*/*w*) of alcohol mixture (ethanol + n-propanol) and 2.5% (*w*/*w*) of phospholipid; the preparation method used was mixing.
- H-yE-xMix-1 denotes a sample prepared with the following composition: 10% (*w*/*w*) of ethylene glycol, 0.625% (*w*/*w*) of surfactant mixture, 30% (*w*/*w*) of ethanol and 2.5% (*w*/*w*) of phospholipid, the preparation method used was homogenization.

### 2.2. Preparation of Ethosomes

In order to compare the stability, as determined by zeta potential measurements, as well as the hydrodynamic diameter (D*_h_*) and polydispersity index of the prepared carriers containing various aliphatic alcohols, their branched analogs, glycols, and surfactants, a series of formulations were prepared, which are presented in the subsections below. Each sample was prepared using the cold method, which included the following steps:

Preparation of Phase I by weighing appropriate amounts of phospholipid, alcohol, and/or glycol and/or surfactant into a tightly sealed vial (see Section 2.3).

Preparation of Phase II by weighing the appropriate amount of water into a tightly sealed vial (see Section 2.3).

Heating both phases to a temperature of 30 °C.

Slow addition of Phase II to Phase I under stirring using a magnetic stirrer (300 rpm, IKA, Staufen, Germany).

Stirring of the resulting solution at 700 rpm for 15 min (except for samples containing n-butyl alcohol (2), which were stirred for 1 min at 700 rpm, followed by 14 min at 1000 rpm) or homogenization for 3 min at 15,000 rpm (IKA, Ultra Turrax^®^ T25 easy clean) for samples designated as H (see Table 2).

**Figure 1:**
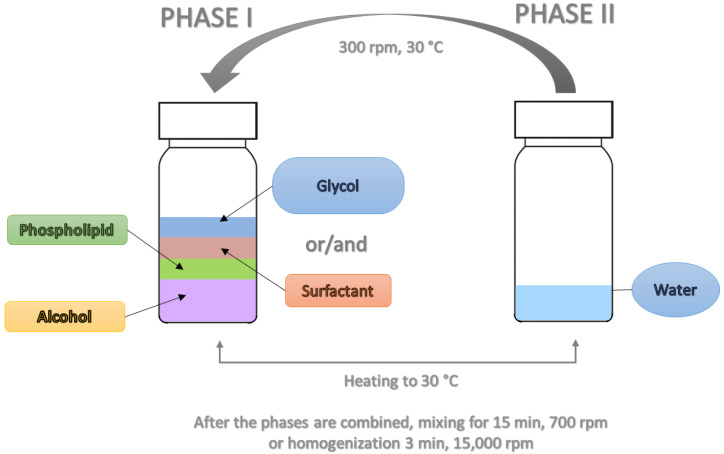
Schematic representation of preparation procedure for obtaining ethosomes.

The preparation procedure is illustrated in Figure 1.

### 2.3. Optimization of Preparation of Ethosomes

**Figure 2:**
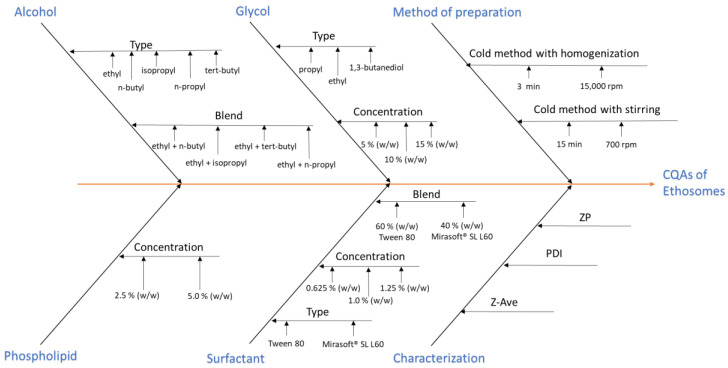
Ishikawa (fishbone) diagram demonstrating Critical Quality Attribute (CQA) of the obtained ethosomes.

This study employed zeta potential (ZP), hydrodynamic diameter (D*_h_*), and polydispersity index (PDI) as key quality attributes for the prepared ethosomes. This aligns with the initial stage of the Quality by Design (QbD) process, which assumes building product quality already at the design stage, specifically the determination of Critical Quality Attributes (CQAs), (Figure 2) [30,31]. Potential substances and processes that may affect the quality of the obtained ethosomes were identified. The most critical variables included phospholipid concentration, the type of alcohol or alcohol mixture, the type and concentration of glycol, the type and concentration of surfactant and their mixtures, and the preparation methods employed. The results demonstrated that the type and concentration of surfactant, glycol, and the preparation method have a significant impact on the quality attributes of ethosomes.

#### 2.3.1. Optimization of Phospholipid Weight Concentration

To optimize the phospholipid weight concentration, formulations containing 2.5% (*w*/*w*) (F1–F5) or 5% (*w*/*w*) (kF1–kF5) of phospholipid were prepared with 30% (*w*/*w*) of alcohols (1–5).

#### 2.3.2. Optimization of Alcohol Type

Formulations were prepared using five different alcohols (30% (*w*/*w*)), namely, ethanol (F1), n-butanol (F2), isopropanol (F3), n-propanol (F4), and tert-butanol (F5). These formulations were then modified by the addition of glycols and surfactants, as described in the sections below. Based on the obtained results, three alcohols, vis. ethanol (1), n-butanol (2), and n-propanol (4), were selected, as giving samples showing the most favorable stability parameters, specifically zeta potential (ZP) with values furthest from zero, indicating higher colloidal stability, mean hydrodynamic diameter (D*_h_*) with the smallest values, and the lowest polydispersity index (PDI).

Additionally, samples containing 2.5% (*w*/*w*) and 5.0% (*w*/*w*) of phospholipid were prepared with different alcohol mixtures (15% (*w*/*w*) of each): ethanol + n-butanol (M(1+2)), ethanol + isopropanol (M(1+3), ethanol + n-propanol (M(1+4), and ethanol + tert-butanol (M(1+5). The formulations containing 2.5% (*w*/*w*) of phospholipid were subsequently modified with propylene glycol and a surfactant (Tween 80).

#### 2.3.3. Optimization of Glycol Type and Its Concentration

The initial step in optimizing the glycol additive involved assessing its impact on ethosomes stability, maintaining consistent phospholipid concentrations. Therefore, samples containing 5% (*w*/*w*) of phospholipid, 10% (*w*/*w*) of propylene glycol, and 30% (*w*/*w*) of alcohol (kP1–kP5) were prepared. Ethosomes were also prepared with 2.5% (*w*/*w*) of phospholipid, 10% (*w*/*w*) of propylene glycol, and binary mixtures of alcohols (15% *w*/*w* each): ethanol + n-butanol (P-M(1+2)), ethanol + isopropanol (P-M(1+3)), ethanol + n-propanol (P-M(1+4)), and ethanol + tert-butanol (P-M(1+5)).

Next, to select the appropriate glycol type, formulations were prepared with 2.5% (*w*/*w*) of phospholipid, 10% (*w*/*w*) of either propylene glycol (P), ethylene glycol (E), or 1,3-butanediol (B), and 30% (*w*/*w*) of one of the following alcohols: ethanol (P1), n-butanol (P2), isopropanol (P3), n-propanol (P4), or tert-butanol (P5), along with 0.625% (*w*/*w*) of Tween 80 surfactant (8T).

Following the selection of ethylene glycol as the optimal glycol type, its concentration was further optimized. Samples containing 5% (*w*/*w*) (xE-x8T-(1–5)), 10% (*w*/*w*) (yE-x8T-(1–5)), and 15% (*w*/*w*) (zE-x8T-(1–5)) of ethylene glycol, as well as 2.5% (*w*/*w*) of phospholipid, 30% (*w*/*w*) of alcohol, and 0.625% (*w*/*w*) of Tween 80 were prepared.

#### 2.3.4. Optimization of Surfactant Presence

After optimizing the phospholipid concentration, as well as the type and concentration of glycol, together with selecting three alcohols (ethanol (1), n-butanol (2), and n-propanol (4)), surfactant selection was also undertaken. To optimize the surfactant concentration and evaluate the effects of different surfactants on the prepared carriers, samples were prepared containing: 30% (*w*/*w*) of selected alcohols, 2.5% (*w*/*w*) of phospholipid, 10% (*w*/*w*) of ethylene glycol, and surfactant at the following concentrations: 0.625% (*w*/*w*) (25% of total phospholipid content), 1.00% (*w*/*w*) (40% of total phospholipid content), 1.25% (*w*/*w*) (50% of total phospholipid content). Two surfactants were tested, namely, Tween 80 (Sigma-Aldrich) and Mirasoft^®^ SL L60 (Syensqo). Additionally, formulations were prepared using surfactant mixtures, in which 40% (*w*/*w*) of the total surfactant content was the biosurfactant Mirasoft^®^ SL L60, and 60% (*w*/*w*) was Tween 80. The total surfactant concentrations used in the mixtures were as follows: 0.625% (*w*/*w*) (yE-xMix-1), 1.00% (*w*/*w*) (yE-yMix-1), and 1.25% (*w*/*w*) (yE-zMix-1).

All of the above formulations described in Section 2.3.4 were prepared using two different methods in order to optimize ethosomes preparation process: (i) cold method with stirring (15 min; 700 rpm) and (ii) cold method without stirring, followed by homogenization (3 min; 15,000 rpm).

### 2.4. Stability Testing

To assess the stability of the prepared samples, the following measurements were performed: zeta potential (ZP) was assessed using Electrophoretic Light Scattering (ELS), and we determined mean hydrodynamic diameter (D*_h_*) and polydispersity index (PDI) using Dynamic Light Scattering (DLS).

#### 2.4.1. Zeta Potential (ZP)

Zeta potential was measured using a Zetasizer Nano-Z (Malvern, UK) via the ELS technique, applying Phase Analysis Light Scattering (PALS) mode, which detects small phase shifts in scattered light caused by moving particles [32]. Measurements were performed on the day of sample preparation, one day after preparation, and after 14 days of refrigerated storage. Each measurement was conducted in triplicate, and mean values with standard deviation were calculated. Before each analysis, 30 μL of the sample was diluted in 15 mL of water. Finally, the analyte concentration was 0.002 mL of solvent, the measurement temperature was 25 °C, the scattering angle was 173°, and the number of replicates (n) was 3. Based on the results obtained, the standard deviation was calculated (±SD).

#### 2.4.2. Hydrodynamic Diameter (D*_h_*) and PDI Values

The mean hydrodynamic diameter (D*_h_*) was measured using the DLS technique with a Zetasizer Nano-Z (Malvern) instrument. DLS measures time-dependent fluctuations in the light scattered by a suspension of nanoparticles, as determined by their Brownian motion [33,34].

It is generally accepted that a polydispersity index (PDI) below 0.1 indicates a mono-disperse system. Values in the range of 0.1–0.7 correspond to a system approaching monodispersity, whereas PDI above 0.7 indicates a polydisperse system, characterized by a broad particle size distribution in the solution [35]. A PDI value greater than 0.3 was considered as the threshold for acceptable stability.

The Zetasizer Nano Z apparatus utilizes the patented NIBS (Non-Invasive Back Scatter) technique, with a detector positioned at a 173° angle. This setup provides optimal sensitivity while permitting measurements at relatively high sample concentrations. Alternative scattering angles, including forward scattering, were not employed due to the instrument’s design specifications. Polystyrene cuvettes (10 × 10 × 45 mm, Sarstedt, Hildesheim, Germany) were used. Measurements were taken on the day of sample preparation, but also one day and 14 days after refrigerated storage. Each measurement included three replicates, from which the mean value and standard deviation were calculated. Before each analysis, 30 μL of the sample was diluted in 15 mL of water. Finally, the analyte concentration was 0.002 mL/mL of solvent, the measurement temperature was 25 °C.

#### 2.4.3. Statistical Analyses

**Scheme 1:**
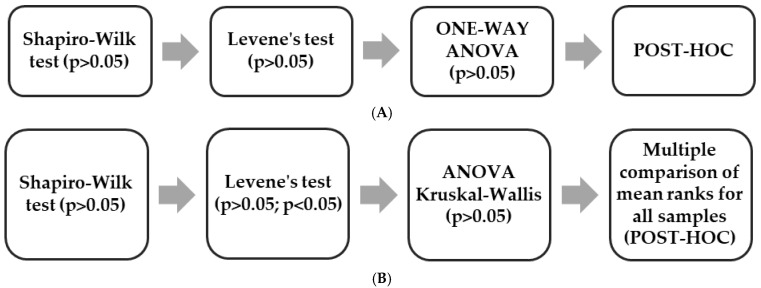
Selection of appropriate statistical tests: (**A**) One-way Anova; (**B**) Anova Kruskal-Wallis.

Statistical analyses were performed using *Statistica 13* (TIBCO Software Inc., San Ramon, CA, USA). Depending on the distribution and characteristics of the data, either one-way analysis of variance (one-way ANOVA) or the Kruskal–Wallis test (rank-based ANOVA) was applied. When statistically significant differences were detected (*p* < 0.05), appropriate post hoc procedures were conducted: ANOVA post hoc tests for parametric data or the *Multiple comparison of mean ranks for all samples* procedure following the Kruskal–Wallis test for non-parametric data. The selection of statistical tests was based on the dataset structure presented in Scheme 1.

The one-way ANOVA was performed for the results shown in Figure 6, while the Kruskal–Wallis test was applied to all remaining datasets. The *Multiple comparison of mean ranks for all samples* post hoc analysis was conducted for the data presented in Figure 3, Figure 4 and Figure 5 and Figures 7–12 and in Table 3 and Table 4.

### 2.5. SEM

Images of selected samples were taken using a scanning electron microscope SEM FEI Quanta 250FEG (Thermo Fisher Scientific, Hillsboro, OR, USA). Samples (20 µL of each) were drop-cast onto silicon wafer substrates and allowed to dry at room temperature without coating. SEM analyses were conducted under high vacuum using an accelerating voltage of 5 kV. The histograms showing the range of particle diameters were generated using the ImageJ 1.54g software Java 1.8.0_345 (64-bit). All histograms were generated from quantitative measurements obtained from two representative images of each sample.

## 3. Results and Discussion

### 3.1. Optimization of Phospholipid Weight Concentration

**Figure 3:**
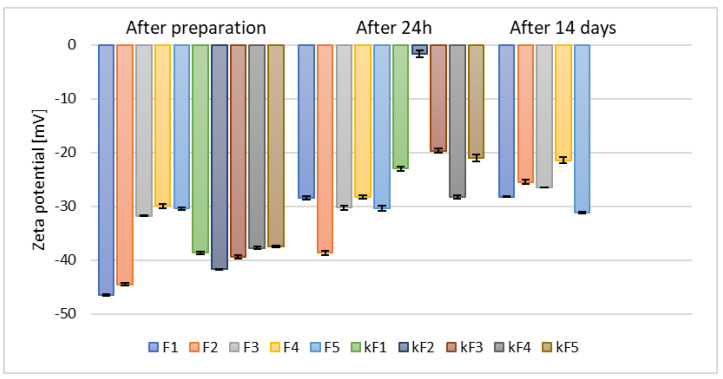
The results of zeta potential measurements for samples prepared with 30% (*w*/*w*) of different alcohols and containing 2.5% (*w*/*w*) (F1–F5) and 5% (*w*/*w*) (kF1–kF5) of phospholipid (*p* = 0.0012) (names of the samples according to Table 2).

Following the preparation of ethosomes containing 2.5% (*w*/*w*) of phospholipid and 30% (*w*/*w*) of alcohol, zeta potential measurements conducted after 14 days of refrigerated storage yielded values ranging from −21.47 mV to −31.17 mV (Figure 3).

The greater the deviation of the zeta potential from zero, the higher the potential stability of the sample [36]. Doubling the phospholipid concentration to 5% (*w*/*w*) resulted in significantly lower zeta potential values as early as 24 h after sample preparation (kF1–kF5). The least stable sample was the one based on n-butanol (kF2), for which the zeta potential measured after 24 h reached only −1.70 mV. Consequently, based on the zeta potential measurements taken after 24 h and considering the higher cost associated with the increased amount of raw material, the samples containing 5% (*w*/*w*) of phospholipid (kF1–kF5) were excluded from further analysis.

Our results are in good agreement with previous studies by Yang et al., who demonstrated that the optimal phospholipid concentration for ethosomes prepared with 30% (*v*/*v*) ethanol was 2.45% (*w*/*v*), as higher phospholipid concentrations led to an unfavorable increase in vesicle size [37]. In contrast, a research group from Iraq that prepared ethosomes containing 30% (*v*/*v*) ethanol and either 2% (*w*/*w*) or 3% (*w*/*w*) phospholipid, and reported slightly more favorable zeta potential values (~−40.0 mV) [38], compared to the results presented in this study. However, ethosomes prepared by a research group from Thailand [39], prepared with concentrations of ethanol and water identical with those applied during our studies, along with 3% (*w*/*w*) or 6% (*w*/*w*) of phospholipid and supplemented with 0.5% (*w*/*w*) of cholesterol, exhibited zeta potential values similar to those of our sample F1, namely after 14 days storage >−32 and −34 mV (Figure 3).

### 3.2. Optimization of Alcohol Type

Ethosomes, in contrast to the conventional liposomes, have been reported to enhance permeation of the drug through stratum corneum and promote subsequent retention of the active compounds within the skin layers [7,40]. This enhancement is primally attributed to the incorporation of alcohol—specifically ethanol—into the carrier structure. Ethanol functions as an edge activator and promotes fluidization of the highly ordered lipophilic structure of the stratum corneum, thereby facilitating drug permeation. Additionally, the presence of ethanol influences the physicochemical properties of ethosomes, for example, by lowering the phase transition temperature of the lipids forming the vesicle membrane [14].

Zeta potential measurements for samples prepared with a single type of alcohol and varying phospholipid concentrations were presented in the previous section (Figure 3). These results confirmed that ethanol led to the formation of the most stable ethosomal formulations. However, measurements also showed that the stability of ethanol-based samples (F1) significantly decreased after 24 h and 14 days of storage. In contrast, samples prepared with tert-butyl alcohol (F5) exhibited a slightly different behavior–although their initial zeta potential values were lower compared to ethanol-based samples, they remained stable even after 14 days of storage.

**Figure 4:**
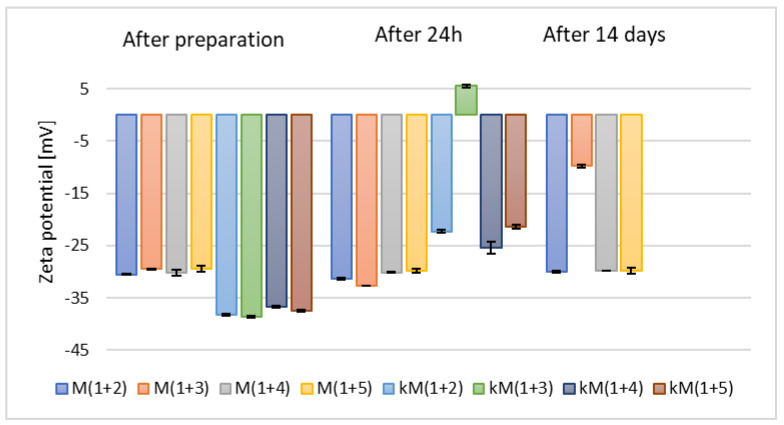
The results of zeta potential measurements for samples containing 2.5% (*w*/*w*) (M(1+(2–5))) and 5% (*w*/*w*) (kM(1+(2–5))) of phospholipid and mixtures of alcohols (*p* = 0.0872) (names of the samples according to Table 2).

Ethanol is a well-known permeation enhancer that interacts with the hydrophilic sites of the stratum corneum lipid bilayer and enhances lipid fluidity. Moreover, it plays an important role in defining zeta potential, average size, stability, and entrapment efficiency of ethosomes [41]. Nevertheless, based on the results presented in Figure 3 and previous studies by Yang et al. [42], alternative alcohols were also investigated in our study. It has been established that alcohols other than ethanol can also function effectively as skin permeation enhancers, due to their ability to modify the lipid-based layer of the stratum corneum [43]. Consequently, samples containing selected alcohol mixtures (as listed in Table 2) with 2.5% (*w*/*w*) (M(1+(2–5))) and 5% (*w*/*w*) (kM(1+(2–5))) phospholipid content were prepared. These formulations exhibited a similar trend in zeta potential measurements relative to alcohol type as samples based on single alcohols (F1–F5), as shown in Figure 4.

Additionally, it was observed that these samples demonstrated lower stability compared to those based on individual alcohols (F1–kF5). Based on the zeta potential measurements taken after 24 h and due to the increased costs associated with the use of a higher amount of raw material, the samples containing 5% (*w*/*w*) of phospholipid (kM(1+(2–5))) were excluded from further analysis.

Among the selected alcohols, ethanol has the shortest chain and is moderately polar, whereas isopropanol is more hydrophobic, which influences the fluidity of the lipid membrane and may contribute to its destabilization [44]. N-propanol and n-butanol possess longer carbon chains, which weaken hydrogen bonding in ethosomes. Their extended alkyl chains reduce polarity, limit the formation of stable hydrogen bonds, and penetrate deeper into the lipid layer, thereby loosening the membrane structure [8]. Tert-butanol is less polar than ethanol but more polar than pure lipids, allowing it to modify the lipid bilayer structure of ethosomes. At a concentration of 30% (*w*/*w*), tert-butanol can decrease the zeta potential by disrupting phospholipid organization or enhancing solvent penetration into the bilayer, reducing the effective surface charge [45]. Moreover, tert-butanol can enhance system homogeneity by stabilizing the formation of lipid vesicles and lowering the interfacial tension between ethanol and water phases. However, at concentrations above 15% (*w*/*w*), it may induce system destabilization and vesicle aggregation. Additionally, tert-butanol can reduce the hydrodynamic diameter of vesicles by promoting a more compact and flexible lipid structure during ethosome formation. Excessive amounts may lead to loosening of the lipid membrane and the formation of larger, unstable structures [46].

The selection of alcohols was continued in subsequent stages of the optimization process in ethosomes preparation.

### 3.3. Optimization of Glycol Type and Its Concentration

Glycols are recognized as well-known penetration enhancers. In particular, polyethylene glycol (PG) is characterized by low toxicity, reduced skin irritation, and higher viscosity and hygroscopicity compared to ethanol, as well as enhancing formulation stability. This combination of properties increases the affinity of the active compounds for the dermis and supports their retention in the deeper layers of the skin [47]. It has been shown that the combination of ethanol with other alcohols, including glycols, results in binary ethosomes with smaller vesicle sizes, leading to the improved stability and reduced tendency for aggregation. In addition, binary ethosomes exhibit higher entrapment efficiency and enhanced skin permeation. However, it is crucial to properly adjust the ethanol-to-PG ratio in order to optimize drug permeation [48]. Therefore, our study also included the optimization of both the type and concentration of glycol, as these parameters may influence the stability of the resulting ethosomes. The types of glycols used in this study are listed in Table 2.

**Figure 5:**
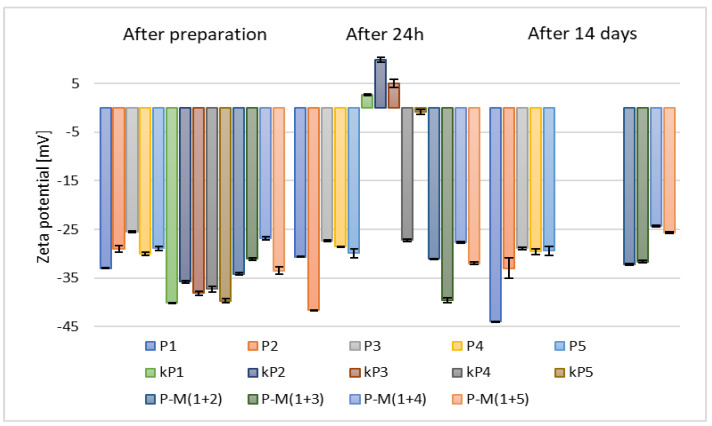
The results of zeta potential measurements for samples containing 2.5% (*w*/*w*) (P1–P5) and 5% (*w*/*w*) (kP1–kP5) of phospholipid and 10% (*w*/*w*) of propylene glycol (p = 0.0301) (names of the samples according to Table 2).

The results of zeta potential measurements (Figure 5) for samples containing 2.5% (*w*/*w*) of phospholipid, 30% (*w*/*w*) of alcohol, and 10% (*w*/*w*) of propylene glycol (P1–P5) indicated higher stability compared to those prepared with 5% (*w*/*w*) of phospholipid (kP1–kP5).

Consequently, based on the zeta potential measurements taken after 24 h and considering the increased costs associated with the use of larger amounts of raw materials, the samples containing 5% (*w*/*w*) of phospholipid (kP1–kP5) were excluded from further analysis. In comparison to ethosomes prepared without the addition of glycol (F1–F5) (Figure 3), the stability of the carriers containing propylene glycol, 2.5% (*w*/*w*) of phospholipid, and 30% (*w*/*w*) of alcohol (P1–P5) was higher, with zeta potential values after two weeks ranging from –28.9 mV to –44 mV. The addition of glycol increases the viscosity of the aqueous phase, thereby reducing collisions between vesicles. In addition, glycols form hydrogen bonds with the phospholipid heads, further stabilizing the lipid bilayer [9].

Furthermore, the addition of glycol reduces surface tension and facilitates the formation of homogeneous bubbles.

Samples based on alcohol mixtures and 2.5% (*w*/*w*) of phospholipid (M(1+(2–5))) exhibited lower stability than those prepared with individual alcohols (P1–P5) (Figure 5), but higher stability than the samples without propylene glycol (F1–F5) (Figure 3). All zeta potential results obtained during our study for samples containing propylene glycol were more favorable than those reported by the research group of Zhang, who studied various ratios of ethanol and propylene glycol. Their reported values ranged from –25.7 mV to –9.83 mV [49].

The addition of glycol increases the zeta potential. Glycol can strongly influence surface charge in phospholipid systems [50]. It can also penetrate the phospholipid headgroups and alter the order and polarity of the interfacial layer, thereby affecting charge distribution and the resulting shear (zeta) potential. Moreover, glycol may interact with ethanol, further enhancing the surface charge values [9,49].

**Figure 6:**
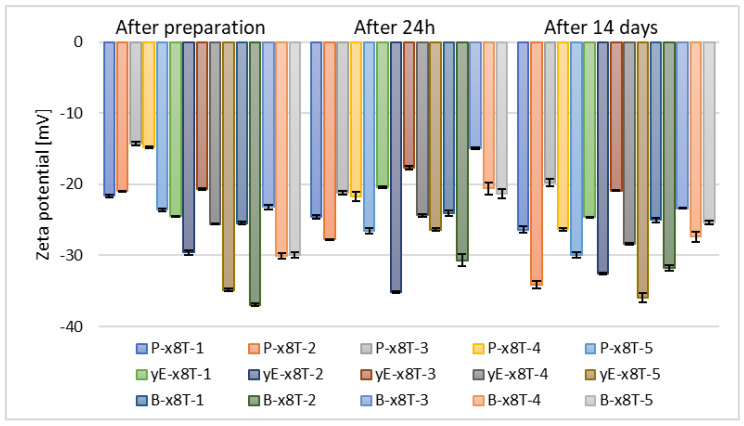
Zeta potential measurement results for samples containing 2.5% (*w*/*w*) of phospholipid, 30% (*w*/*w*) of alcohol, 0.625% (*w*/*w*) of Tween 80 (x8T) and 10% (*w*/*w*) of glycol (*p* = 0.5858) (names of the samples according to Table 2).

Subsequently, after selecting the appropriate phospholipid concentration, another set of samples was prepared, namely those containing 10% (*w*/*w*) of various glycols, such as propylene glycol (P-x8T-(1–5)), ethylene glycol (yE-x8T-(1–5)), and 1,3-butanediol (B-x8T-(1–5)), also in combination with 0.625% (*w*/*w*) of the Tween 80 surfactant. All carriers exhibited similar zeta potential values after 14 days of refrigerated storage (Figure 6).

It can be observed that samples based on propylene glycol and 1,3-butanediol show notable variations in zeta potential values over time. Ultimately, after 14 days of storage, the zeta potential ranged from –34.13 mV to –19.77 mV for propylene glycol, from –31.77 mV to –25.03 mV for 1,3-butanediol, and from –35.93 mV to –20.88 mV for ethylene glycol, as illustrated in Figure 6. The results of zeta potential measurements obtained for the sample containing ethanol and Tween 80 indicate its higher stability compared to carriers containing the same raw materials used in different proportions, as reported by the research group of Ahad, where the highest recorded ZP value was in the range of –6.19 ± 0.10 mV [51]. The type of glycol influences the zeta potential of ethosomes. Small glycols (e.g., propylene glycol, ethylene glycol, 1,3-butanediol, and glycerol) interact with the polar groups of phospholipids, modifying the local dielectric environment, thereby altering the charge distribution and zeta potential [49,52,53].

**Figure 7:**
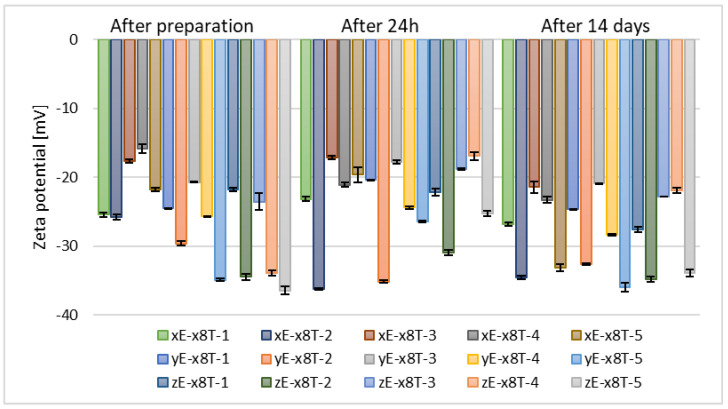
Zeta potential measurement results for samples containing 2.5% (*w*/*w*) of phospholipid, 30% (*w*/*w*) of alcohol, 0.625% (*w*/*w*) of Tween 80, and 5% (*w*/*w*) (xE-x8T-(1–5)), 10% (*w*/*w*) (yE-x8T-(1–5)), and 15% (*w*/*w*) (zE-x8T-(1–5)) of ethylene glycol (*p* = 0.0163) (names of the samples according to Table 2).

The next stage in optimizing the preparation parameters of ethosomes involved selecting the appropriate concentration of the chosen glycol, namely ethylene glycol. Samples containing this glycol exhibited the most optimal stability based on zeta potential measurements, meaning the values deviated furthest from zero. After 14 days of refrigerated storage, the zeta potential values for the tested alcohols, vis. ethanol, n-butanol, and n-propanol, were comparable across all concentrations of ethylene glycol. However, for the two remaining alcohols used (n-propanol and tert-butanol), the zeta potential values after 14 days of samples storage were higher for formulations containing 10% (*w*/*w*) of ethylene glycol (yE-x8T-(1–5)), as shown in Figure 6 and Figure 7.

The concentration of glycol has a significant impact on the zeta potential of ethosomes. An Egyptian research group demonstrated that glycerol concentrations ranging from 10% (*w*/*v*) to 30% (*w*/*v*) influence both particle size and zeta potential, with glycerosomes generally exhibiting a distinct and often more negative—zeta potential compared to control liposomes without glycol [52]. Consistent observations were reported by an Italian research group, confirming the modulatory effect of glycerol concentration on zeta potential [54]. To date, no studies have been published investigating the influence of ethylene glycol concentration on the zeta potential of ethosomal systems.

### 3.4. Optimization of Surfactant Presence

Transethosomes usually show greater skin permeation compared to classical ethosome-based formulations, since they contain surfactants that act as permeation enhancers or edge activators [55,56]. Vesicles of transethosomes are also characterized by smaller size, enhanced elasticity and deformability. This is most likely due to a synergistic effect between ethanol and the surfactant, which promotes rearrangement of the lipid bilayer in transethosomes [56]. To determine the influence of surfactants on the ethosomes prepared in our study, two different types of surface-active agents were employed, namely Tween 80 and the biosurfactant Mirasoft^®^ SL L60, which is derived from natural ingredients via fermentation using natural yeast.

Depending on their structure, surfactants exert varying effects on the zeta potential value, while generally enhancing the overall stability of the resulting ethosomes. Surfactants may penetrate the lipid bilayer or adsorb onto its surface, thereby altering the surface charge density, influencing the polarization of the diffusion layer, and ultimately modulating the zeta potential [57,58].

**Figure 8:**
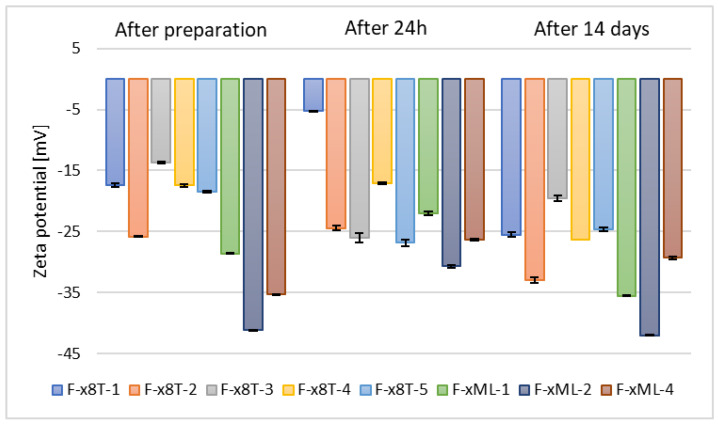
Zeta potential measurement results for samples containing 2.5% (*w*/*w*) phospholipid, 30% (*w*/*w*) alcohol, 0.625% (*w*/*w*) Tween 80 (F-x8T-(1–5)) or Mirasoft SL L60 (F-xML-(1/2/4)) (*p* = 0.0555) (names of the samples according to Table 2).

It was observed that samples containing surfactant, specifically 0.625% (*w*/*w*) of Tween 80, and 30% (*w*/*w*) of alcohol (F-x8T-(1–5)) exhibited slightly lower stability compared to their counterparts in the form of classical ethosomes (F1–F5), and significantly lower stability compared to ethosomes formulated with propylene glycol (P1–P5). The zeta potential values for these samples ranged from –19.6 mV to –32.97 mV, as shown in Figure 8.

In contrast, the zeta potential measurements for samples based on selected alcohols (ethanol, n-butanol, and n-propanol) and biosurfactant Mirasoft^®^ SL L60 (F-xML-1, F-xML-2, F-xML-4) ranged from –29.33 mV to –42 mV. Based on the zeta potential values, the stability of F-xML-1, F-xML-2, F-xML-4 samples can be considered comparable to that of ethosomes containing ethylene glycol and based on individual alcohols (P1–P5). Tween 80 and Mirasoft^®^ SL L60 biosurfactant (a sophorolipid in lactone form), both non-ionic surfactants, form a steric layer that masks the electrostatic charge of ethosome particles, thereby reducing the zeta potential. Simultaneously, they enhance stability by forming a protective layer that prevents particle aggregation [51,59].

Particles of 500 Da can penetrate the epidermis [60]. Deep penetration into the epidermis/dermis is possible for particles ≤100 nm in size, with low PDI and good elasticity [61,62]. For local surface action or accumulation in the hair follicle, sizes of 100–300 nm may be sufficient and sometimes advantageous due to the larger loads of active substance [57,60].

**Figure 9:**
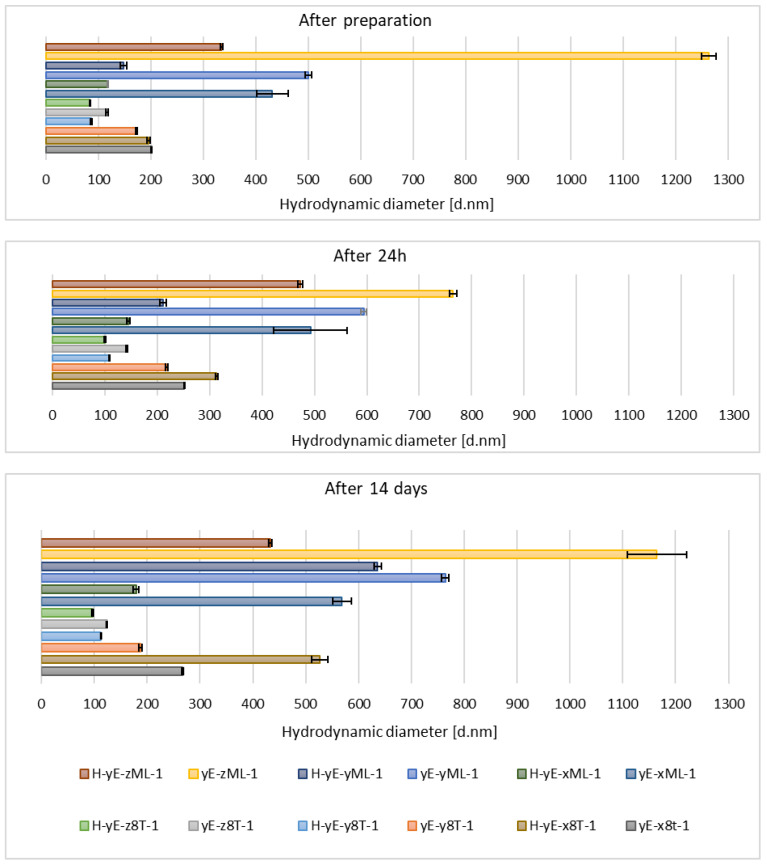
Hydrodynamic diameter measured for samples containing different concentrations of surfactants (0.625 (x), 1.0 (y), 1.25 (z) % (*w*/*w*)), namely Tween 80 (8T) and Mirasoft^®^ SL L60 (ML), 10% (*w*/*w*) of ethylene glycol (yE) and prepared by two methods comprising only stirring and subjected to homogenization (H) (*p* = 0.0498) (names of the samples according to Table 2).

**Figure 10:**
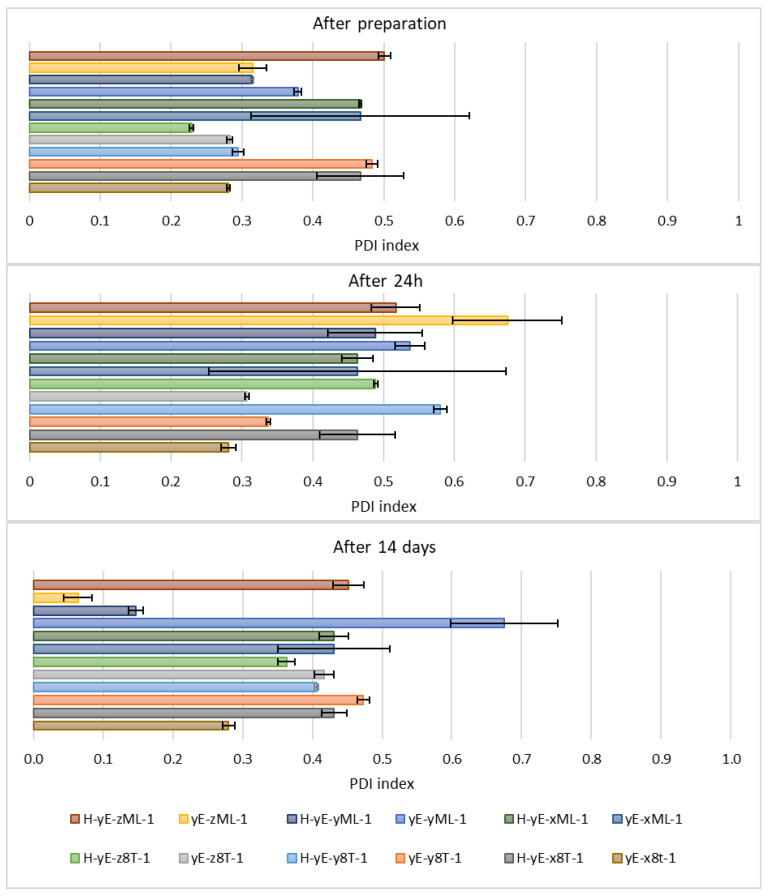
Polydispersity index values measured for samples containing different concentrations of surfactants (0.625 (x), 1.0 (y), 1.25 (z) % (*w*/*w*)), namely Tween 80 (8T) and Mirasoft^®^ SL L60 (ML), 10% (*w*/*w*) ethylene glycol (yE) and prepared by two methods comprising only stirring and subjected to homogenization (H) (*p* = 0.015) (names of the samples according to Table 2).

Immediately after sample preparation and on the following day, the hydrodynamic diameter of samples containing Tween 80 that were not subjected to homogenization was equal to or smaller than that of the homogenized samples. However, the polydispersity index (PDI) was higher in the homogenized samples. The zeta potential results were comparable during the first two measurements. After 14 days, for samples containing Tween 80, homogenization, which was intended to reduce hydrodynamic diameter and thereby stabilize the carriers, resulted in both an increase in D*_h_* and PDI in the sample containing 0.625% (*w*/*w*) of surfactant (H-yE-x8T-1), and a decrease in both parameters for the samples with higher surfactant concentrations (H-yE-y8T-1, H-yE-z8T-1) (Figure 9 and Figure 10). The zeta potential results were comparable between the non-homogenized samples (yE-x8T-1, yE-y8T-1, yE-z8T-1) and those subjected to homogenization (Table 3).

**Table 3:** Zeta potential measurement results for samples containing 2.5% (*w*/*w*) of phospholipid, 30% (*w*/*w*) of ethanol, 10% (*w*/*w*) of ethylene glycol, and three different concentrations of surfactants (0.625, 1.0, 1.25% (*w*/*w*)), namely Tween 80 and Mirasoft^®^ SL L60; (*p* = 0.0003) (names of the samples according to Table 2).

	Directly After Preparation		After 24 h in the Refrigerator		After 14 Days in the Refrigerator	
Formulation	Zeta Potential [mV]	±SD [mV]	Zeta Potential [mV]	±SD [mV]	Zeta Potential [mV]	±SD [mV]
yE-x8T-1	−25.70	0.44	−24.97	0.74	−27.90	0.52
yE-y8T-1	−26.27	0.15	−24.17	0.49	−29.70	0.17
yE-z8T-1	−26.50	0.10	−25.23	0.15	−30.13	0.31
yE-xML-1	−26.07	0.25	−25.50	0.20	−26.73	0.15
yE-yML-1	−32.77	0.12	−26.30	0.23	−21.40	0.17
yE-zML-1	−31.63	0.21	−26.40	0.26	−32.20	0.61
H-yE-x8T-1	−26.60	0.10	−21.73	0.21	−27.83	0.32
H-yE-y8T-1	−35.33	0.38	−29.97	0.55	−29.20	0.26
H-yE-z8T-1	−26.80	0.10	−30.87	0.12	−28.97	0.25
H-yE-xML-1	−19.43	0.91	−29.20	0.10	−30.00	0.70
H-yE-yML-1	−35.37	0.15	−19.10	0.17	−26.53	0.25
H-yE-zML-1	−27.27	0.12	−26.43	0.21	−28.33	0.21

On the other hand, the hydrodynamic diameter of samples containing biosurfactant and subjected to homogenization, measured immediately after preparation and on the following day, was nearly two times smaller than that of the non-homogenized samples. During this period, the PDI was comparable for samples with the lowest biosurfactant concentration. However, at the highest concentration of Mirasoft^®^ SL L60, the homogenized samples exhibited a higher PDI upon preparation and a lower PDI after 24 h, compared to the non-homogenized counterparts. After preparation, the zeta potential values were more favorable, i.e., further from zero, for non-homogenized samples. However, 24 h after preparation, the zeta potential values for both homogenized and non-homogenized samples were comparable. After 14 days of refrigerated storage, samples subjected to homogenization resulted in reduced hydrodynamic diameter (Figure 9) and improved zeta potential values (Table 3) for samples containing biosurfactant. The PDI of the sample with 0.625% (*w*/*w*) Mirasoft^®^ SL L60 (H-yE-xML-1) remained comparable to the non-homogenized counterpart, while significantly decreased for the sample with 1.00% (*w*/*w*) biosurfactant (H-yE-yML-1) and meaningfully increased for the sample with 1.25% (*w*/*w*) biosurfactant (H-yE-zML-1) subjected to homogenization (Figure 10). The zeta potential values were more favorable, i.e., further from zero, for homogenized samples containing 0.625% (*w*/*w*) and 1.00% (*w*/*w*) of biosurfactant, compared to the non-homogenized samples (yE-xML-1, yE-yML-1, yE-zML-1). However, the sample containing 1.25% (*w*/*w*) of this surfactant exhibited a less favorable zeta potential value after homogenization (Table 3).

Immediately after preparation and 24 h later, the hydrodynamic diameter was smaller for all non-homogenized samples compared to those that underwent homogenization. The polydispersity index (PDI) was significantly lower immediately after preparation for the homogenized sample containing the lowest concentration of surfactants, but after 24 h, this difference diminished. The remaining samples exhibited the same trend upon preparation, while after 24 h, the results for homogenized and non-homogenized samples were comparable. The zeta potential values were similar for both preparation methods, namely with or without the homogenization step. Homogenized samples containing different amounts of surfactant mixtures, after 14 days of storage in a refrigerator, exhibited more favorable zeta potential values (i.e., further from zero) at concentrations of 1.00% (*w*/*w*) (H-yE-yMix-1) and 1.25% (*w*/*w*) (H-yE-zMix-1). For the lowest concentration (H-yE-xMix-1, yE-xMix-1), the zeta potential remained comparable (Table 4).

**Table 4:** Zeta potential measurement results for samples containing 2.5% (*w*/*w*) of phospholipid, 30% (*w*/*w*) of ethanol, 10% (*w*/*w*) of ethylene glycol, and mixtures of surfactants, namely Tween 80 and Mirasoft^®^ SL L60 (total concentration: 0.625, 1.0, 1.25% (*w*/*w*) where 40% (*w*/*w*) was Mirasoft^®^ SL L60, and 60% (*w*/*w*) was Tween 80) (*p* = 0.0025) (names of the samples according to Table 2).

	Directly After Preparation		After 24 h in the Refrigerator		After 14 Days in the Refrigerator	
Formulation	Zeta Potential [mV]	±SD [mV]	Zeta Potential [mV]	±SD [mV]	Zeta Potential [mV]	±SD [mV]
yE-xMix-1	−28.30	0.10	−20.50	0.10	−24.53	0.25
yE-yMix-1	−28.47	0.40	−26.57	0.31	−23.73	0.06
yE-zMix-1	−27.87	0.06	−28.57	0.12	−27.20	0.10
H-yE-xMix-1	−20.67	0.21	−17.27	0.42	−23.47	0.40
H-yE-yMix-1	−30.77	0.25	−31.40	0.46	−37.97	0.35
H-yE-zMix-1	−28.37	0.21	−27.33	0.06	−30.03	0.06

**Figure 11:**
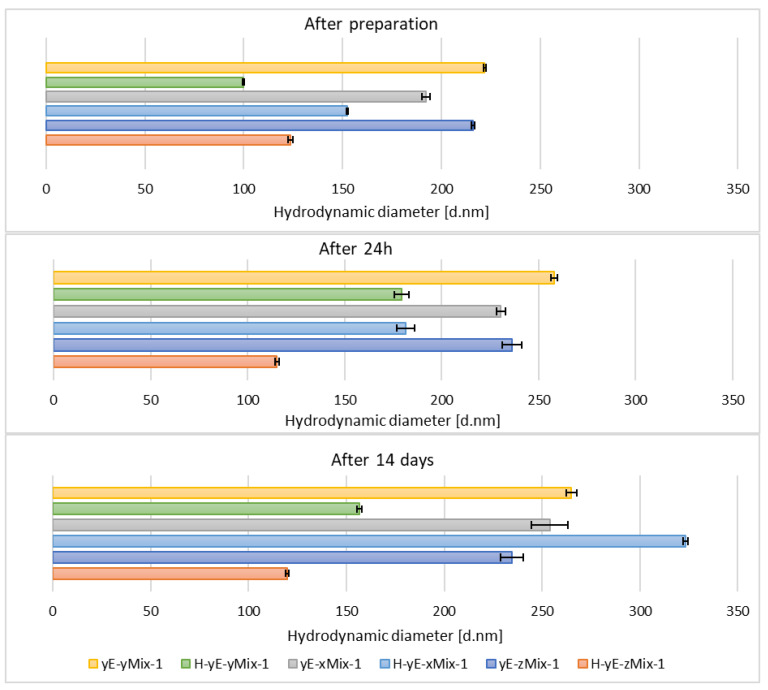
Hydrodynamic diameter measured for samples containing different concentrations of mixtures (Mix) of surfactants, namely Tween 80 (8T) and Mirasoft^®^ SL L60 (ML) (total concentrations: 0.625 (x), 1.0 (y), 1.25 (z) % (*w*/*w*), where 40% (*w*/*w*) was Mirasoft^®^ SL L60, and 60% (*w*/*w*) was Tween 80), 10% (*w*/*w*) ethylene glycol (yE) and prepared by two methods comprising only stirring and subjected to homogenization (H) (*p* = 0.1353) (names of the samples according to Table 2).

The hydrodynamic diameter significantly decreased for homogenized samples containing the lowest and highest surfactant concentrations of Tween 80 and Mirasoft^®^ SL L60, while the sample containing 1.00% (*w*/*w*) of surfactant exhibited a notable increase in the hydrodynamic diameter after homogenization (Figure 11), which may indicate particle aggregation. It shows that the presence of the biosurfactant Mirasoft^®^ SL L60 helps to obtain stable ethosomes with lower surfactant concentrations compared to its synthetic counterpart.

**Figure 12:**
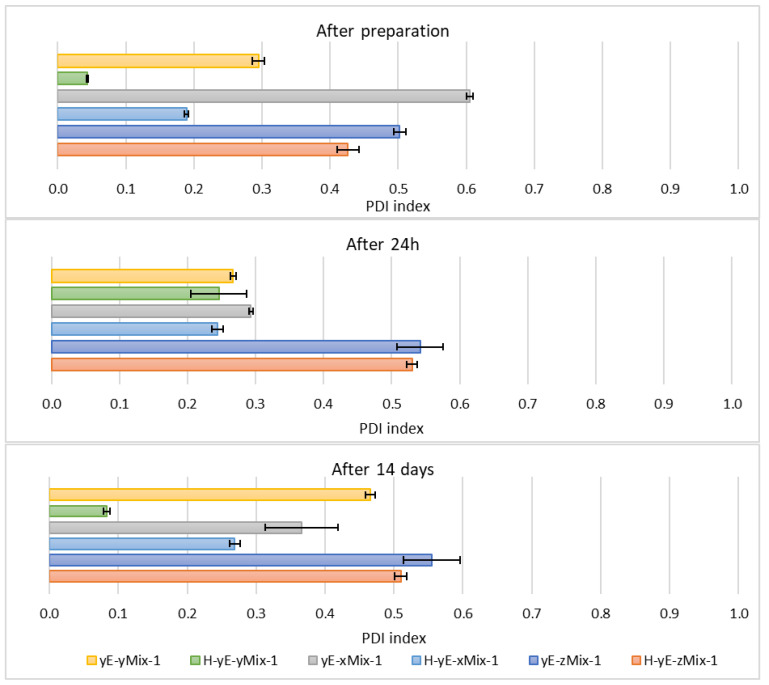
Polydispersity index values measured for samples containing different concentrations of mixtures (Mix) of surfactants, namely Tween 80 (8T) and Mirasoft^®^ SL L60 (ML) (total concentrations: 0.625 (x), 1.0 (y), 1.25 (z) % (*w*/*w*), where 40% (*w*/*w*) was Mirasoft^®^ SL L60, and 60% (*w*/*w*) was Tween 80), 10% (*w*/*w*) ethylene glycol (yE) and prepared by two methods comprising only stirring and subjected to homogenization (H) (*p* = 0.1353) (names of the samples according to Table 2).

This is most evident for the sample yE-z8T-1 prepared with 1.25% (*w*/*w*) of Tween 80, for which the ZP, PDI and Z-Ave values after 14 days of storage were −27.90 mV, 0.28, and 267 nm, respectively (Table 3, Figure 9 and Figure 10) and for the sample yE-xMix-1 prepared with 0.625% (*w*/*w*) of a Tween 80 and Mirasoft^®^ SL L60 mixture, for which measurements yielded similar values, namely −24.53 mV, 0.366, and 254 nm for ZP, PDI and Z-Ave, respectively (Table 4, Figure 11 and Figure 12).

Other homogenized samples also demonstrated a decrease in PDI values, indicating greater stability of the formulations studied (Figure 12), as the lower the PDI value, the greater the potential stability of the sample. According to the manufacturers of DLS instruments, a sample can be considered monodispersed when the polydispersity index (PDI) is less than 0.1, moderately polydispersed when the PDI is between 0.1 and 0.4, and polydispersed when the PDI exceeds value of 0.4 [34]. Nonionic surfactants, as previously described, form a steric layer that prevents vesicle aggregation while simultaneously reduce surface tension and modulate the lipid bilayer, either by stiffening or plasticizing it. This effect can lead to a decreased hydrodynamic diameter compared to vesicles lacking nonionic surfactants. However, excessively high surfactant concentrations may induce instability, vesicle coalescence, or micelle formation, ultimately resulting in an increase in particle size [40,63,64,65,66].

Some samples exhibit noticeable changes in their hydrodynamic diameter and zeta potential after 14 days. This may be related to particle aggregation, which subsequently stabilizes and then breaks down into smaller particles [21]. It is also possible that during storage, ethanol evaporates, causing the lipid bilayers to absorb water, which increases D*_h_*. The lipids then reorganize into smaller, stable vesicles, resulting in D*_h_* values below their initial measurements [37].

### 3.5. SEM Measurements

Among the final modifications regarding the selection of the optimal weight concentration of the surfactant mixture (Tween 80 + Mirasoft^®^ SL L60), samples containing the most favorable surfactants concentration were selected, and the influence of the two preparation methods on the shape and size of the obtained ethosomes was compared. For this purpose, the samples were analyzed using a scanning electron microscope. Based on previously described measurements of zeta potential, hydrodynamic diameter, and polydispersity index, the most optimal concentration was identified in samples containing 1.25% (*w*/*w*) of the surfactant mixture (Tween 80 and Mirasoft^®^ SL L60), 2.50% (*w*/*w*) of phospholipid, 30.00% (*w*/*w*) of ethanol, and 10.00% (*w*/*w*) of ethylene glycol. Therefore these samples were subjected to SEM analysis.

**Figure 13:**
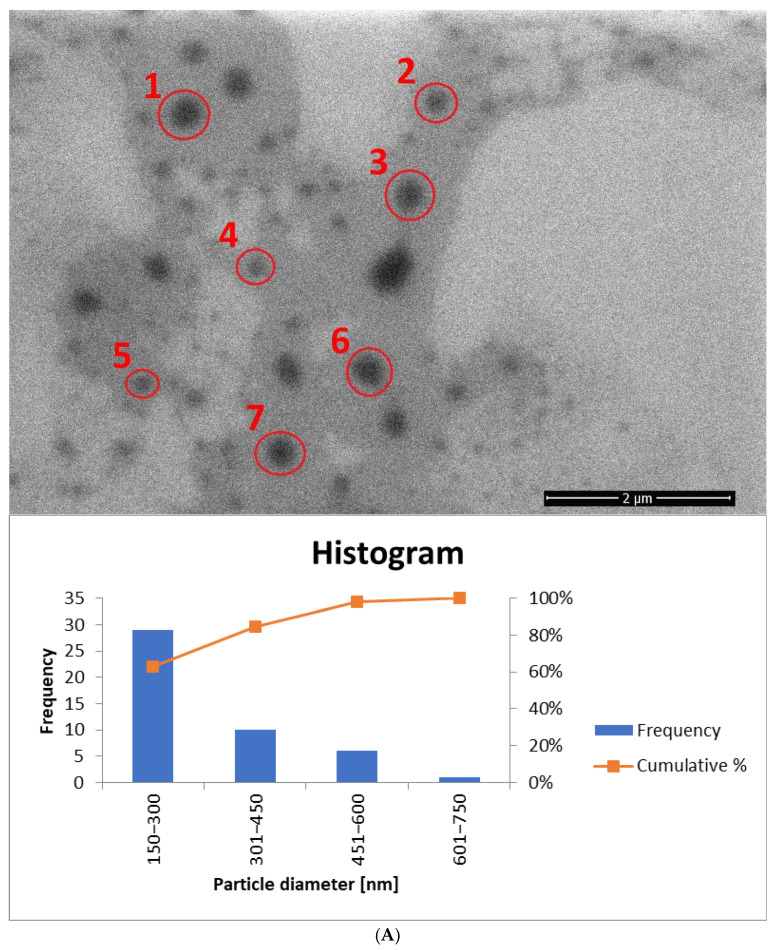

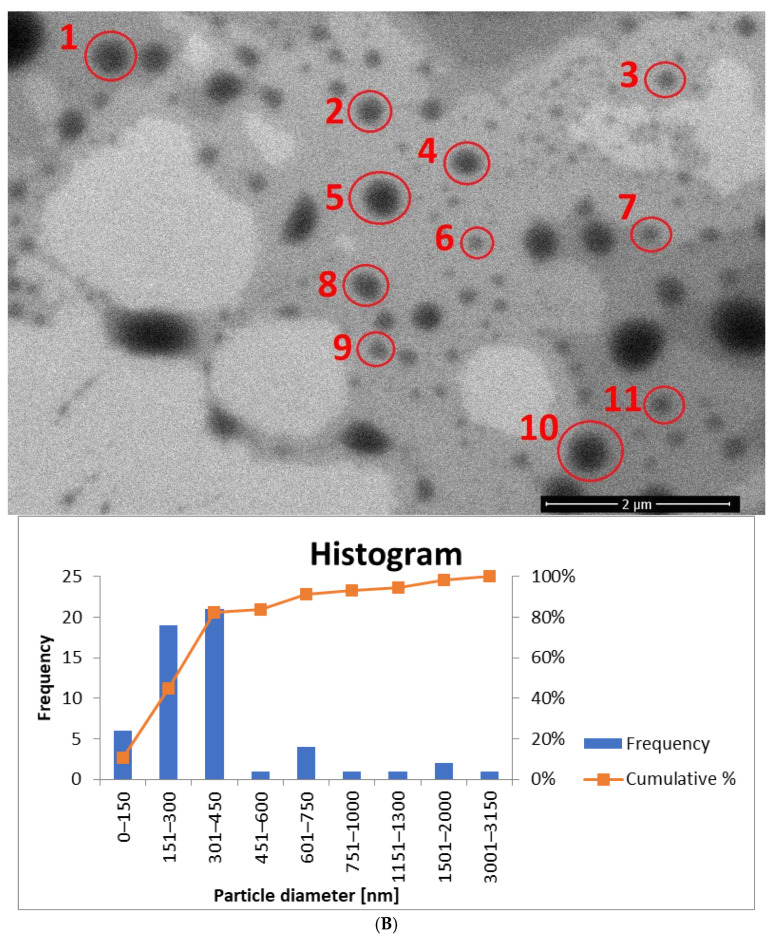

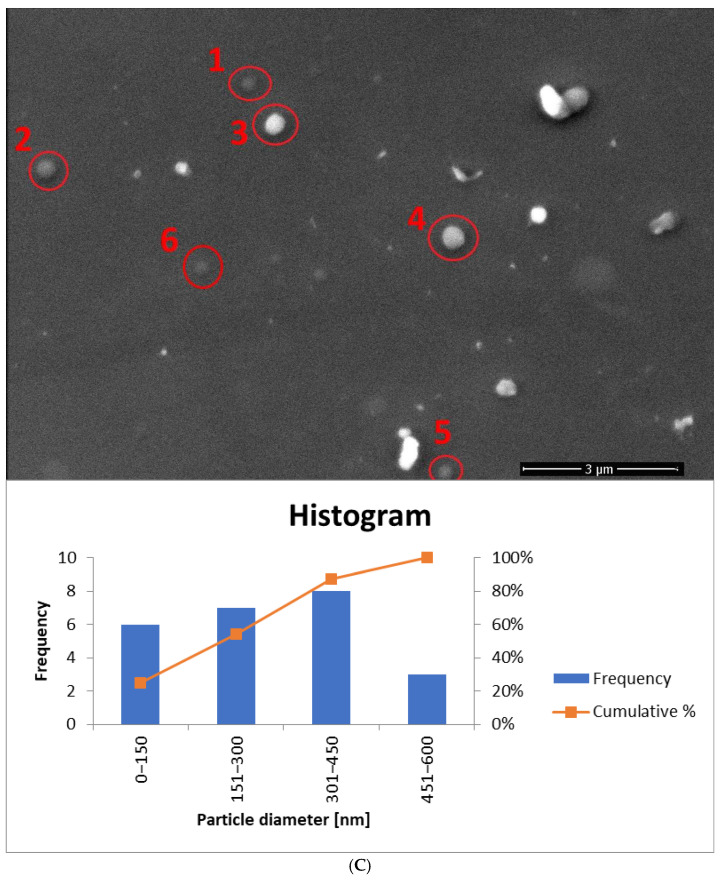

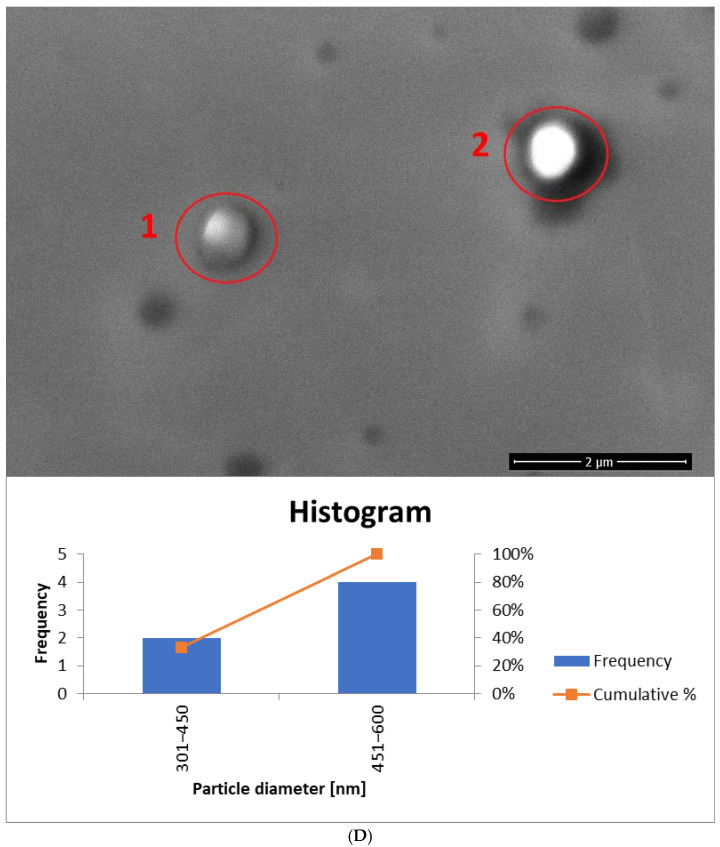
SEM images (accelerating voltage of 5 kV) showing ethosomes in a sample based on: (**A**) 1.25% (*w*/*w*) of Tween 80 and prepared by cold stirring method (yE-z8T-1). Magnification used: 50,000 times; histogram showing the particle diameter, based on (**A**) and Figure S1. (**B**) 1.25% (*w*/*w*) of Tween 80 and prepared by cold stirring method with a homogenization (H-yE-z8T-1). Magnification used: 50,000 times; histogram showing the particle diameter, based on (**B**) and Figure S2. (**C**) 1.25% (*w*/*w*) of Mirasoft® SL L60 and prepared by cold stirring method (yE-zML-1). Magnification used: 30,000 times; histogram showing the particle diameter, based on (**C**) and Figure S3. (**D**) 1.25% (*w*/*w*) of Mirasoft® SL L60 and prepared by cold stirring method with a homogenization (H-yE-zML-1). Magnification used: 50,000 times; histogram showing the particle diameter, based on (**D**) and Figure S4 (names of the samples according to Table 2).

In the SEM images, spherical structures corresponding to individual ethosomes can be observed (Figure 13A,B). The particles in the samples based on Tween 80 surfactant, prepared both with homogenization and without homogenization, exhibit similar sizes, which confirms the results of the previous DLS analysis (Figure 9).

In contrast, the images of the biosurfactant-based samples reveal larger particles. In particular, the sample subjected to homogenization (Figure 13C,D) exhibits larger particle sizes compared to the sample containing Tween 80 and also subjected to homogenization (Figure 13B). Additionally, in the samples based on Mirasoft^®^ SL L60 (Figure 13C,D), spherical structures are significantly fewer in number than in the Tween 80-based samples and are unevenly distributed.

**Figure 14:**
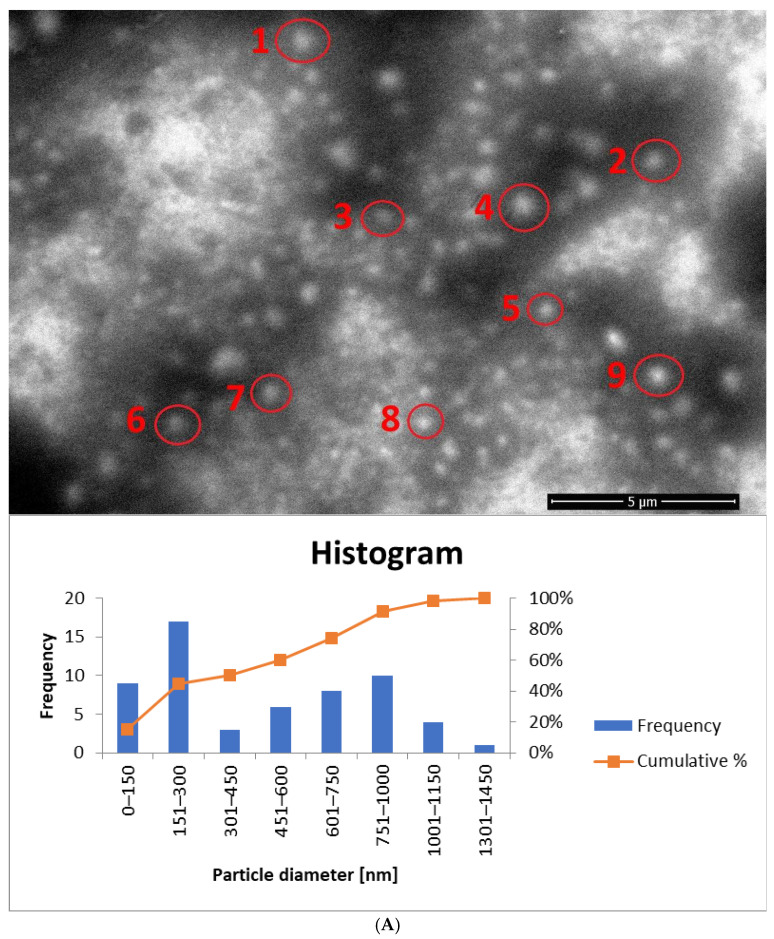

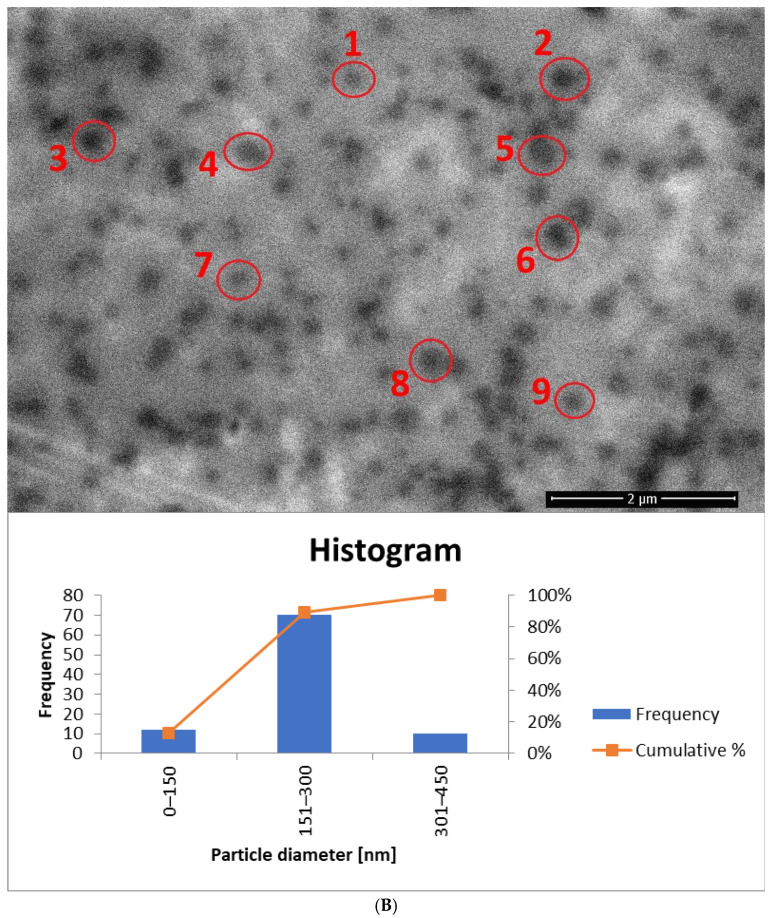
SEM images (accelerating voltage of 5 kV) showing ethosomes in a sample based on: (**A**) 0.625% (*w*/*w*) of the surfactant mixture and prepared by cold stirring method (yE-xMix-1). Magnification used: 20,000 times; histogram showing the particle diameter, based on (**A**) and Figure S5. (**B**) 0.625% (*w*/*w*) of the surfactant mixture and prepared by cold stirring method with a homogenization (H-yE-xMix-1). Magnification used: 50,000 times; histogram showing the particle diameter, based on (**B**) and Figure S6 (names of the samples according to Table 2).

SEM images were also taken for samples containing a mixture of surfactants (Tween 80 + Mirasoft^®^ SL L60) at a concentration of 0.625% (*w*/*w*), which were prepared using two methods: cold processing combined with stirring, and cold processing with homogenization instead of stirring. Both images show a large number of spherical structures. It can be observed that the particles in the sample prepared by cold processing with stirring (Figure 14A) have a larger diameter than those subjected to homogenization (Figure 14B). It was also confirmed during DLS studies (Figure 11). As can be seen, the use of a mixture with a biosurfactant (Figure 14A) allowed not only reduce the total concentration of surfactant used but also to reduce the average particle size compared to a sample containing only Tween 80 (Figure 13A). In addition, the homogenization of samples based on the surfactant mixture (Figure 14B) influenced the reduction in the average particle size relative to the sample prepared by the cold method only with mixing (Figure 14A).

The histogram for sample yE-z8T-1 (Figure 13A) indicates that the Tween 80-based sample contains the highest number of particles with diameters ranging from 150 nm to 300 nm. The average particle diameter determined from SEM images (Figure 13A and Figure S1) is approximately twice the hydrodynamic diameter (D*_h_*) measured by DLS (Table 5). The homogenized sample H-yE-z8T-1 (Figure 13B and Figure S2) shows a similar trend, with most particles falling within the 151–300 nm and 301–450 nm ranges.

The biosurfactant-based samples yE-zML-1 and H-yE-zML-1 exhibit larger particle diameters in SEM analysis. In the non-homogenized sample (Figure 13C and Figure S3), most particles fall within the 151–300 nm and 301–450 nm ranges, whereas in the homogenized sample (Figure 13D and Figure S4) the size distribution is broader (301–450 nm and 451–600 nm). The hydrodynamic diameter measurements, in contrast, show the opposite trend, indicating a significant decrease in particle size after homogenization (Table 5).

For samples prepared with a surfactant mixture, most particles fall within the 151–300 nm range, with SEM-based averages exceeding the DLS results (D*_h_*) (Figure 14A and Figure S5, Table 5). According to SEM measurements, the non-homogenized sample (yE-xMix-1) contains larger particles (up to 1450 nm), whereas in the homogenized sample (H-yE-xMix-1) this range is reduced (Figure 14B and Figure S6). Furthermore, in the latter, the SEM-based average matches well with the hydrodynamic diameters (D*_h_*) measured by DLS (Table 5).

It is worth noting that the observed differences in particle sizes arise primarily from the measurement techniques employed. DLS measures the hydrodynamic diameter (D*_h_*), including the hydration shell, across the entire sample volume, whereas SEM captures only a selected portion of the sample (the core, without the hydration shell), which may not be fully representative. Additionally, SEM-based averages are associated with large standard deviations due to the limited number of particles visible in each image.

## 4. Conclusions

A series of ethosomes formulations containing various types of alcohols, surfactants, and different concentrations of phospholipid, surfactants, and glycol were developed. The presented results recommend that a combination of 30% (*w*/*w*) of ethanol, 2.5% (*w*/*w*) of phospholipid, 10% (*w*/*w*) of ethylene glycol, and 1.25% (*w*/*w*) (yE-z8T-1 and H-yE-z8T-1) of surfactant enables the formation of the most stable carriers. Ethosomes prepared with Tween 80 demonstrate higher stability, smaller average particle size, and greater monodispersity compared to ethosomes based on the biosurfactant. However, promising results were obtained using a mixture of surfactants, where the total surfactant concentration could be reduced to 0.625% (*w*/*w*) (comprising 60% (*w*/*w*) of Tween 80 and 40% (*w*/*w*) of Mirasoft^®^ SL L60) while maintaining satisfactory values of the stability parameters. This demonstrates the supportive role of the tested biosurfactant, enabling the use of more eco-friendly procedures during ethosomes preparation. Additionally, it can be concluded that subjecting the samples to homogenization (3 min, 15,000 rpm) has a positive effect on the stability of the prepared carriers, expressed as average particle size and polydispersity index.

## Figures and Tables

**Table 1:** Comparison of average hydrodynamic diameter, zeta potential, and PDI values for liposomes, ethosomes, and exosomes [7,10,11,12,13,14,15].

Parameter	Liposomes	Ethosomes	Egzosomes
hydrodynamic diameter (D*_h_*)	Approx. 50 nm–200 nm; depends on ingredients and method of preparation	Approx. 120 nm–400 nm; depends on ingredients	Approx. 80 nm–340 nm
polydispersity index (PDI)	<0.3	Approx. 0.15–0.35	Approx. 0.2–0.4
zeta potential (ZP)	Approx. −5–(−)40 mV	Approx. −30–(−)60 mV	>(−)30 mV

**Table 2:** Legend of the sample designations used.

Symbol	Meaning
1	Ethyl alcohol
2	n-Butyl alcohol
3	Isopropyl alcohol
4	n-Propyl alcohol
5	tert-Butyl alcohol
K	5% (*w*/*w*) phospholipid
F	Phospholipid + respective alcohol
P	10% (*w*/*w*) Propylene glycol
B	10% (*w*/*w*) 1,3-Butanediol
xE	5% (*w*/*w*) Ethylene glycol
yE	10% (*w*/*w*) Ethylene glycol
zE	15% (*w*/*w*) Ethylene glycol
M(1+2)	Ethyl alcohol + n-butyl alcohol
M(1+3)	Ethyl alcohol + isopropyl alcohol
M(1+4)	Ethyl alcohol + n-propyl alcohol
M(1+5)	Ethyl alcohol + tert-butyl alcohol
x8T	0.625% (*w*/*w*) Tween 80
y8T	1.00% (*w*/*w*) Tween 80
z8T	1.25% (*w*/*w*) Tween 80
xML	0.625% (*w*/*w*) Mirasoft^®^ SL L60
yML	1.00% (*w*/*w*) Mirasoft^®^ SL L60
zML	1.25% (*w*/*w*) Mirasoft^®^ SL L60
xMix	0.625% (*w*/*w*) surfactant mixture
yMix	1.00% (*w*/*w*) surfactant mixture
zMix	1.25% (*w*/*w*) surfactant mixture
H	Homogenization: 3 min; 15,000 rpm

**Table 5:** Comparison of the D*_h_* values measured using DLS with the particle diameters obtained from SEM images for samples after 14 days of refrigerated storage.

Sample	DLS (D*_h_*) [nm]	±SD [nm]	SEM (Particle Diameter) [nm]	±SD [nm]
yE-z8T-1	124	1	315	131
H-yE-z8T-1	97	1	451	533
yE-zML-1	1165	56	275	144
H-yE-zML-1	433	3	513	92
yE-xMix-1	254	9	509	335
H-yE-xMix-1	157	1	215	64

## Data Availability

The original contributions presented in this study are included in the article/Supplementary Materials. Further inquiries can be directed to the corresponding author.

## Supplementary Materials

The following supporting information can be downloaded at: https://www.mdpi.com/article/10.3390/ma18235355/s1, Figure S1. SEM image (accelerating voltage of 5 kV) showing ethosomes in a sample based on 1.25% (*w*/*w*) of Tween 80 and prepared by cold stirring method (yE-z8T-1). Magnification used was: 50,000 times (names of the samples according to Table 2). Figure S2. SEM image (accelerating voltage of 5 kV) showing ethosomes in a sample based on 1.25% (*w*/*w*) of Tween 80 and prepared by cold stirring method with a homogenization (H-yE-z8T-1). Magnification used was: 20,000 times (names of the samples according to Table 2). Figure S3. SEM image (accelerating voltage of 5 kV) showing ethosomes in a sample based on 1.25% (*w*/*w*) of Mirasoft^®^ SL L60 and prepared by cold stirring method (yE-zML-1). Magnification used was: 50,000 times (names of the samples according to Table 2). Figure S4. SEM image (accelerating voltage of 5 kV) showing ethosomes in a sample based on 1.25% (*w*/*w*) of Mirasoft^®^ SL L60 and prepared by cold stirring method with a homogenization (H-yE-zML-1). Magnification used was: 20,000 times (names of the samples according to Table 2). Figure S5. SEM image (accelerating voltage of 5 kV) showing ethosomes in a sample based on 0.625% (*w*/*w*) of the surfactant mixture and prepared by cold stirring method (yE-xMix-1). Magnification used was: 50,000 times (names of the samples according to Table 2). Figure S6. SEM image (accelerating voltage of 5 kV) showing ethosomes in a sample based on 0.625% (*w*/*w*) of the surfactant mixture and prepared by cold stirring method with a homogenization (H-yE-xMix-1). Magnification used was: 30,000 times (names of the samples according to Table 2).

## Abbreviations

The following abbreviations are used in this manuscript:

ZP	Zeta potential
PDI	Polydispersity index
D*_h_*	Hydrodynamic diameter
SEM	Scanning electron microscopy
TDDS	Transdermal drug delivery system
CQA	Critical quality attribute
ELS	Electrophoretic light scattering
DLS	Dynamic light scattering
PALS	Phase analysis light scattering
