# Supplementary material for: Ethosome-Based Colloidal Systems for Transdermal Delivery: The Role of Biosurfactant in Enhancing Stability and Efficacy
Source: Materials (Basel). 2025 Nov 27;18(23):5355. doi: 10.3390/ma18235355 (PMC12693398; doi:10.3390/ma18235355)
Supplement: Supplementary file 1 [file materials-18-05355-s001.zip › materials-3971963-supplementary.pdf]

SUPPLEMENTARY MATERIALS

Ethosome-based Colloidal Systems for Transdermal Delivery: The Role of Biosurfactant in Enhancing Stability and Efficacy

Jagoda Chudzińska-Skorupinska[1], Agata Wawrzyńczak[2], Agnieszka Feliczak-Guzik[3]*

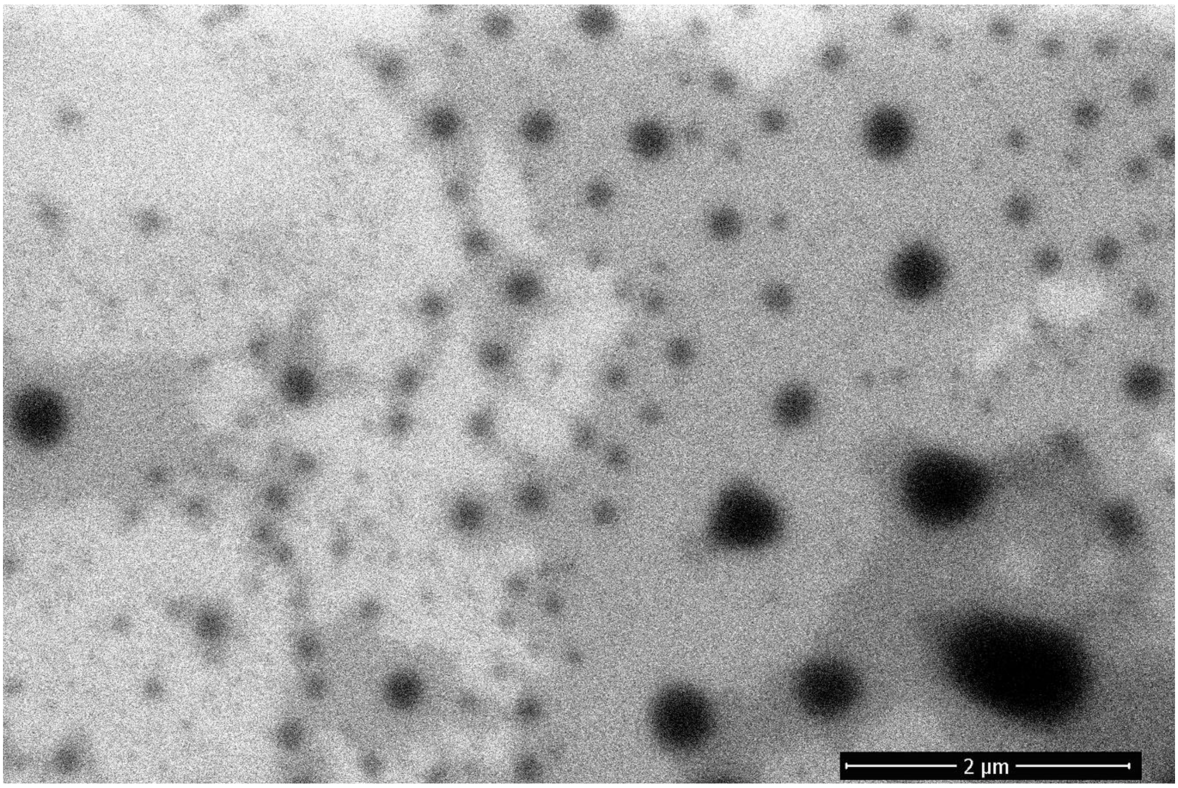

Figure S1. SEM image (accelerating voltage of 5 kV) showing ethosomes in a sample based on 1.25 % (w/w) of Tween 80 and prepared by cold stirring method (yE-z8T-1). Magnification used was: 50,000 times (names of the samples according to Table 2).

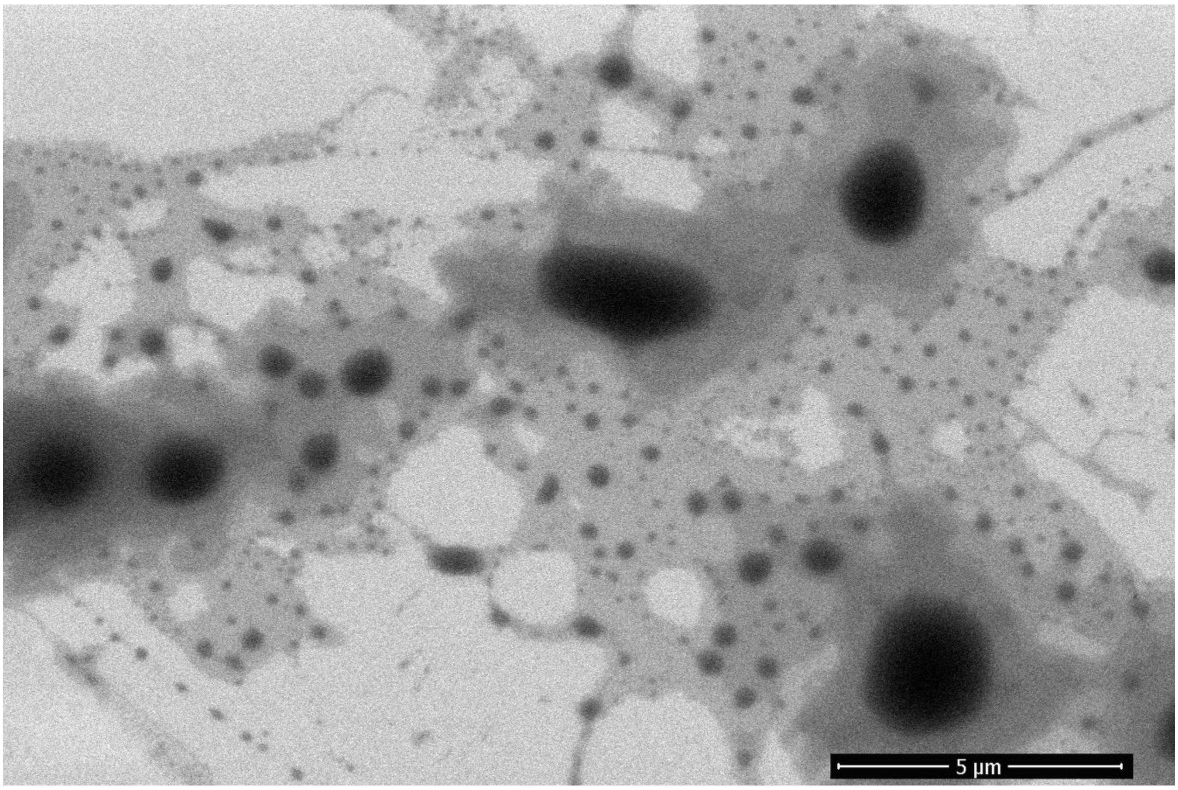

Figure S2. SEM image (accelerating voltage of 5 kV) showing ethosomes in a sample based on 1.25 % (w/w) of Tween 80 and prepared by cold stirring method with a homogenization (H-yE-z8T-1). Magnification used was: 20,000 times (names of the samples according to Table 2).

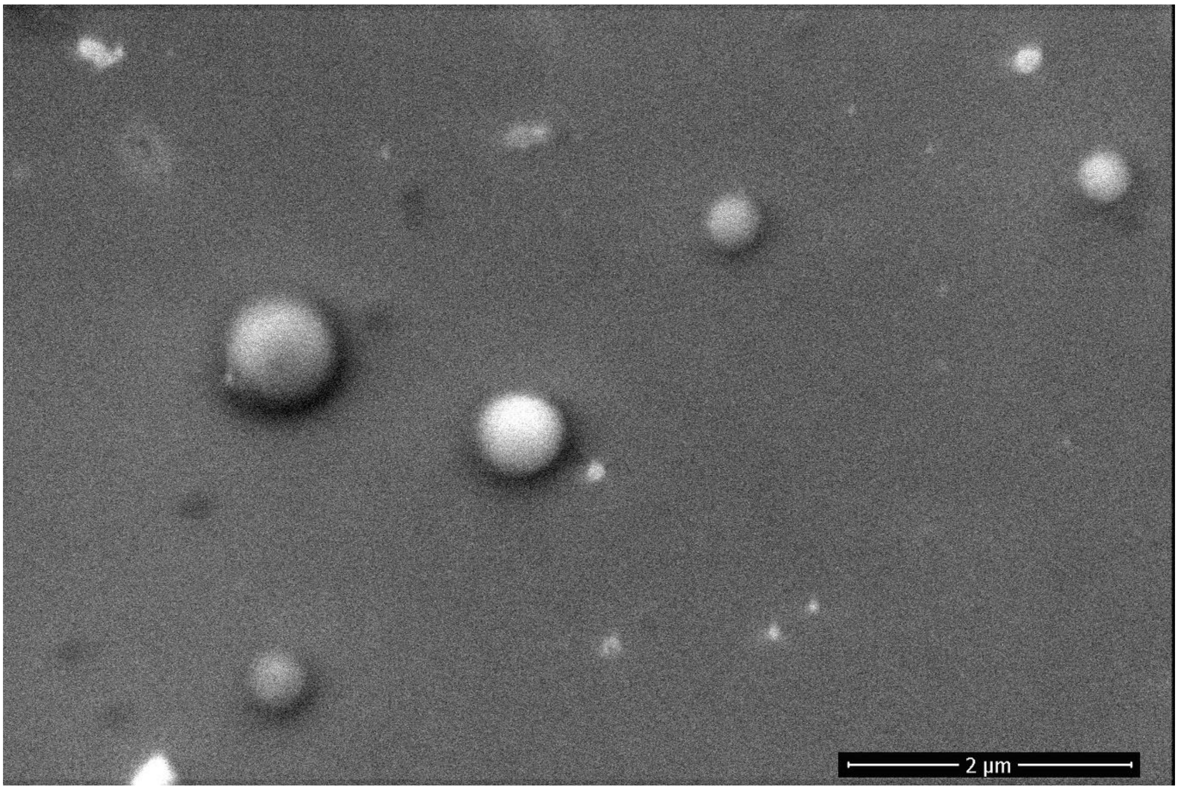

Figure S3. SEM image (accelerating voltage of 5 kV) showing ethosomes in a sample based on 1.25 % (w/w) of Mirasoft® SL L60 and prepared by cold stirring method (yE-zML-1). Magnification used was: 50,000 times (names of the samples according to Table 2).

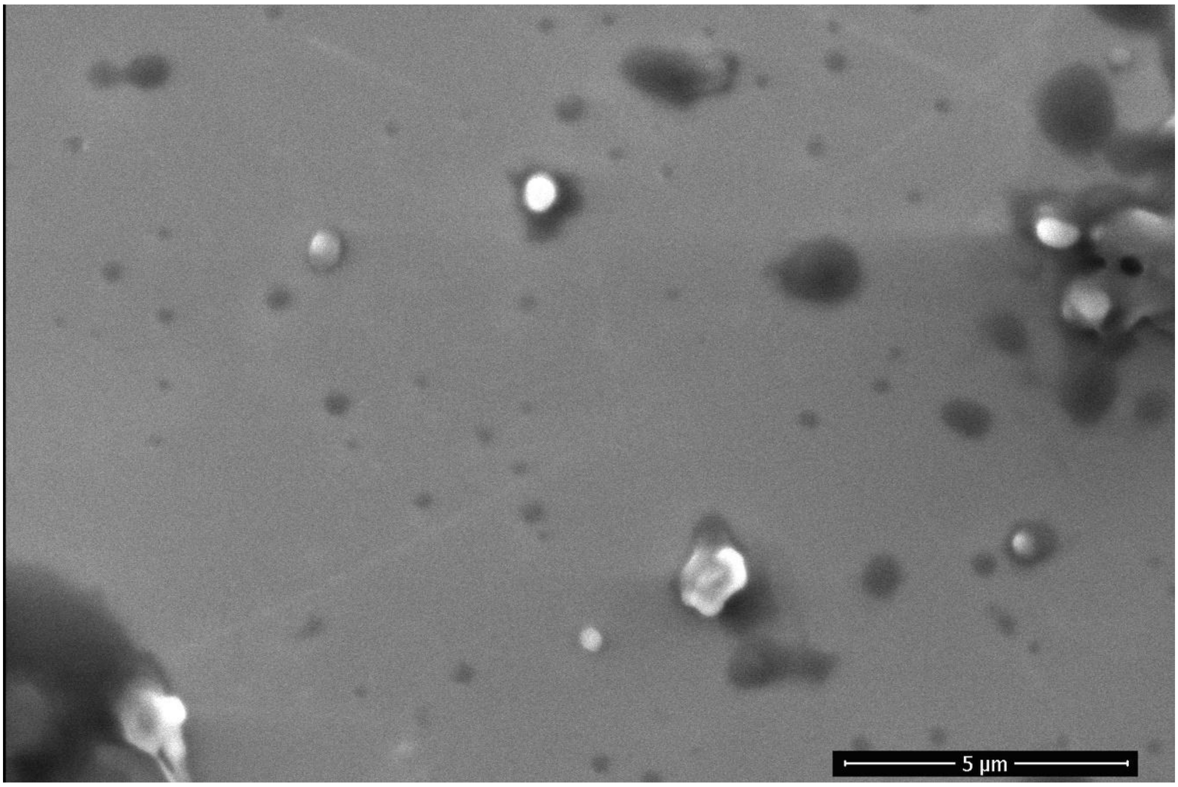

Figure S4. SEM image (accelerating voltage of 5 kV) showing ethosomes in a sample based on 1.25 % (w/w) of Mirasoft® SL L60 and prepared by cold stirring method with a homogenization (H-yE-zML-1). Magnification used was: 20,000 times (names of the samples according to Table 2).

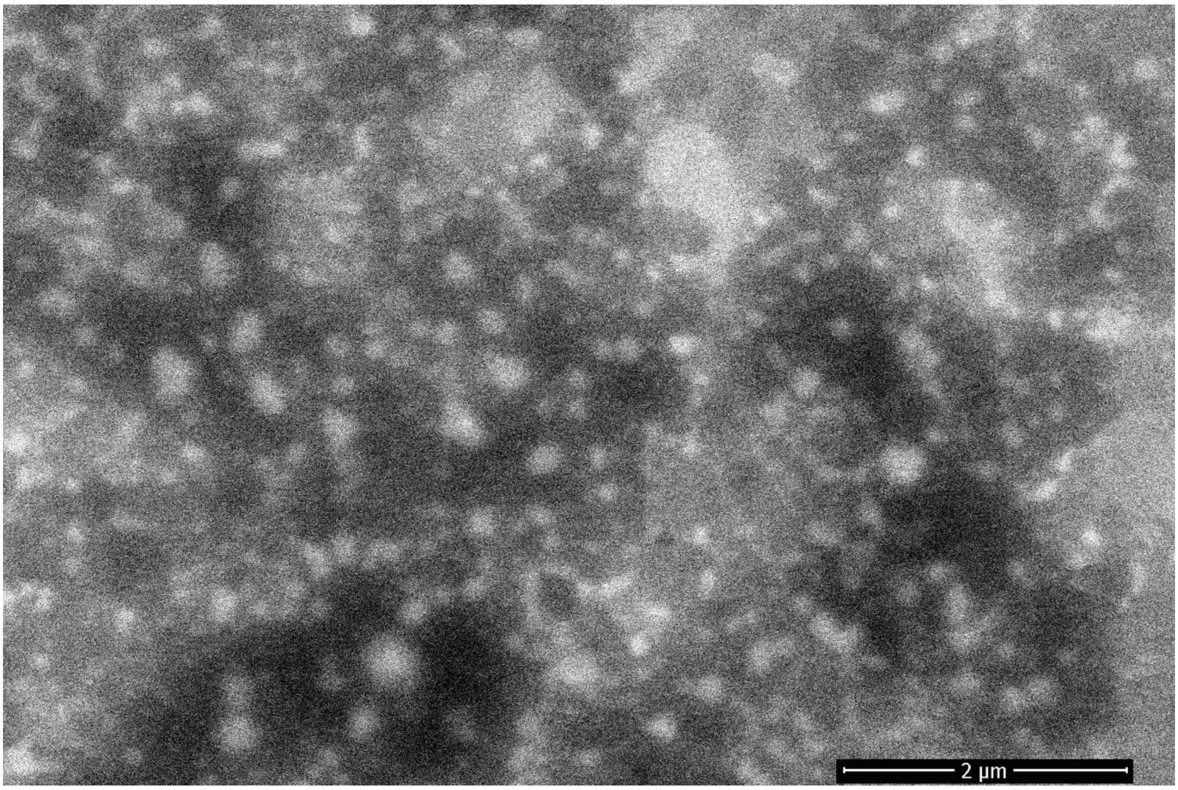

Figure S5. SEM image (accelerating voltage of 5 kV) showing ethosomes in a sample based on 0.625 % (w/w) of the surfactant mixture and prepared by cold stirring method (yE-xMix-1). Magnification used was: 50,000 times (names of the samples according to Table 2).

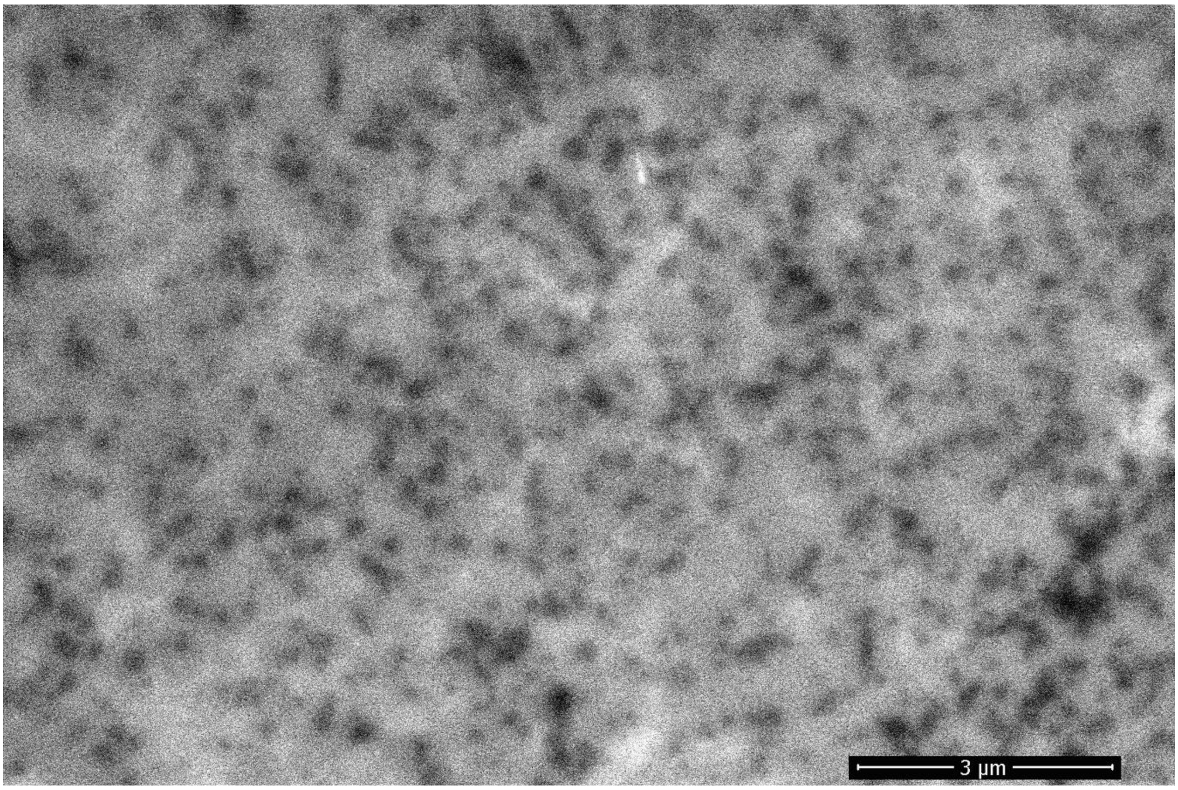

Figure S6. SEM image (accelerating voltage of 5 kV) showing ethosomes in a sample based on 0.625 % (w/w) of the surfactant mixture and prepared by cold stirring method with a homogenization (H-yE-xMix-1). Magnification used was: 30,000 times (names of the samples according to Table 2).
